# Brown adipocytes local response to thyroid hormone is required for adaptive thermogenesis in adult male mice

**DOI:** 10.7554/eLife.81996

**Published:** 2022-11-14

**Authors:** Yanis Zekri, Romain Guyot, Inés Garteizgogeascoa Suñer, Laurence Canaple, Amandine Gautier Stein, Justine Vily Petit, Denise Aubert, Sabine Richard, Frédéric Flamant, Karine Gauthier

**Affiliations:** 1 Institut de Génomique Fonctionnelle de Lyon (IGFL), CNRS UMR 5242, INRAE USC 1370, École Normale Supérieure de Lyon Lyon France; 2 U1213 Nutrition, Diabète et Cerveau, Institut National de la Santé et de la Recherche Médicale Lyon France; Icahn School of Medicine at Mount Sinai United States; Icahn School of Medicine at Mount Sinai United States

**Keywords:** thyroid hormone, brown adipose tissue, energy expenditure, thermogenesis, sequencing, Mouse

## Abstract

Thyroid hormone (T3) and its nuclear receptors (TR) are important regulators of energy expenditure and adaptive thermogenesis, notably through their action in the brown adipose tissue (BAT). However, T3 acts in many other peripheral and central tissues which are also involved in energy expenditure. The general picture of how T3 regulates BAT thermogenesis is currently not fully established, notably due to the absence of extensive omics analyses and the lack of specific mice model. Here, we first used transcriptome and cistrome analyses to establish the list of T3/TR direct target genes in brown adipocytes. We then developed a novel model of transgenic mice, in which T3 signaling is specifically suppressed in brown adipocytes at adult stage. We addressed the capacity of these mice to mount a thermogenic response when challenged by either a cold exposure or a high-fat diet, and analyzed the associated changes in BAT transcriptome. We conclude that T3 plays a crucial role in the thermogenic response of the BAT, controlling the expression of genes involved in lipid and glucose metabolism and regulating BAT proliferation. The resulting picture provides an unprecedented view on the pathways by which T3 activates energy expenditure through an efficient adaptive thermogenesis in the BAT.

## Introduction

Brown adipose tissue (BAT) has the ability to dissipate energy through thermogenesis in response to cold and high-fat diet (HFD), to prevent hypothermia and limit body weight gain, respectively. In response to these stressors, sympathetic nerves stimulate brown adipocytes’ adrenergic receptors to trigger the cAMP-protein kinase A (PKA) (Tabuchi and Sul, 2021), favoring local lipolysis and glycolysis to fuel an increase in energy metabolism (Hankir and Klingenspor, 2018). This is used by the BAT-specific protein UCP1 to favor thermogenesis at the expense of ATP production (Fenzl and Kiefer, 2014). In the long run, this causes an increase in lipogenesis to refill local lipid stocks, mitochondrial biogenesis, and a proliferation of brown adipocytes (Fonseca et al., 2014; Uldry et al., 2006; Fukano et al., 2016). These actions are coordinated by transcription factors, notably involving the coactivator PGC1⍺ (Cao et al., 2004). Importantly, cold exposure induces an indispensable increase expression and activity of the type 2 deiodinase activity (DIO2) resulting in an intracellular conversion of the inactive form of thyroid hormone, thyroxine or T4, into its active form, 3,3′,5-triiodo-L-thyronine or T3 (Fonseca et al., 2014; Silva and Larsen, 1985; Bianco and Silva, 1987).

T3 exerts a large influence on energy expenditure, as hypothyroidism is associated with cold sensitivity and weight gain, while hyperthyroid patients display the opposite phenotype (Bianco and Silva, 1987; Wolf et al., 1996; De Leo et al., 2016; Maushart et al., 2019). T3 binds to the broadly expressed thyroid hormone nuclear receptors, TRα1 and TRβ1/2 (collectively called TR) encoded by the *Thra* and *Thrb* genes (Minakhina et al., 2020). Majority of TR bind to specific response elements resembling the archetypical DR4 consensus sequence (5′AGGTCAnnnnRGGnCA3′) to repress expression of neighboring genes. Upon T3 binding, TR results in a rapid activation of transcription (Flamant, 2016).

T3 concentration drastic increase in BAT during cold exposure (Bianco and Silva, 1988) is crucial, as DIO2*KO* mice have impaired BAT thermogenesis (Schneider et al., 2001). This was notably explained by the ability of T3 to regulate essential thermogenic processes in the BAT-like *Ucp1* expression (Bianco et al., 1988) and fatty acids oxidation (Fonseca et al., 2014). However, the involvement of T3 in energy metabolism is not restricted to brown adipocytes, as it directly increases hepatic lipid and glucose metabolism (Ritter et al., 2020), and favors exercise-associated thermogenesis in the muscle (Nicolaisen et al., 2020). It also triggers white adipose tissue (WAT) browning, a process in which heat-producing ‘beige’ adipocytes expressing *Ucp1* emerge in the WAT (Martínez-Sánchez et al., 2017; Medina-Gomez et al., 2008). Finally, T3 stimulates indirectly BAT thermogenesis by acting in the hypothalamic ventro-medial nuclei where it triggers the sympathetic outflow to BAT (López et al., 2010).

Thus, T3 exerts a broad influence on different tissues to regulate energy expenditure, making the relative importance of BAT thermogenesis in T3-dependent energy expenditure unclear. Moreover, T3 action directly in the BAT has not been exhaustively described as (1) no transcriptomic and cistromic analyses have been led to obtain T3/TR target genes in this tissue and (2) T3 also indirectly controls the BAT through the brain, making the role of local T3 difficult to pinpoint. Here, we aimed at distinguishing these two levels and focus on the T3 cell-autonomous (i.e., local) function in brown adipocytes, and address its importance in adaptive thermogenesis. To address this aim, we combined genomic analysis and the phenotyping of mice with selective blockade of T3 signaling in brown adipocytes. The resulting picture provides an unprecedented view on the pathways by which T3 activates energy expenditure through an efficient adaptive thermogenesis in the BAT.

## Results

### BATKO mice present a BAT-specific deletion of T3 signaling

**Figure 1.**
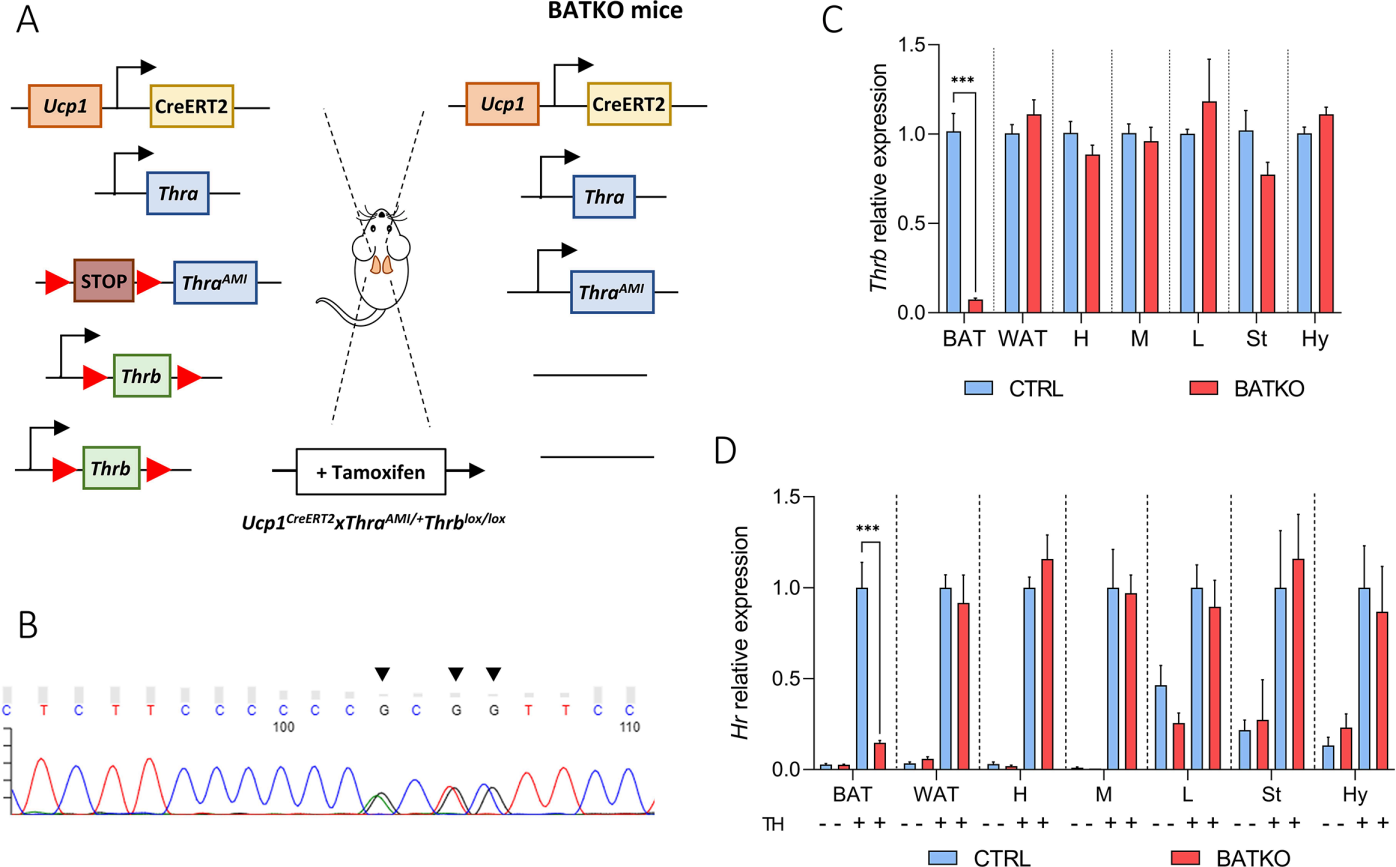
BATKO mice present a brown adipose tissue (BAT)-specific blockade of T3 signaling. (**A**) Schematic representation of the BATKO mice. BATKO mice carry the *Ucp1^CreERT2^* transgene, allowing the brown-adipocyte-specific expression of the tamoxifen-sensitive CreERT2 recombinase. BATKO mice are also heterozygous for the *ThraAMI* allele, which encodes the TRα1L400R dominant-negative receptor after Cre-mediated deletion of a STOP cassette flanked by loxP sequences. BATKO mice are homozygous for the *Thrblox* allele in which exon 3 is flanked by two tandem-arranged loxP sequences. After tamoxifen injection, Cre-mediated recombination selectively excise the loxP-flanked sequences in brown adipocytes, resulting in the expression of TRα1L400R and elimination of TRβ. Control mice (CTRL, not represented here) had the same genotype except for the absence of the *Ucp1^CreERT2^* transgene and were also tamoxifen treated. (**B**) Sanger sequencing chromatogram of a fragment of *Thra* cDNA prepared from BAT RNA of BATKO mice. Arrows indicate the positions of the TRα1L400R mutations. (**C**) Relative mRNA expression of *Thrb* in different peripheral and central tissues of CTRL and BATKO mice (H: heart, M: muscle, L: liver, St: striatum, Hy: hypothalamus) (*n* = 5–6/group). (**D**) Evaluation of T3 response in propylthiouracil (PTU)-fed CTRL and BATKO mice through the induction of *Hr*, a well characterized TR target gene, after 24 hr of TH (+ or −; *n* = 5–7/group). Statistical significance is shown for the comparison of CTRL and BATKO mice treated with TH. Error bars represent the standard deviation (SD). ***p < 0.001 for the indicated comparisons.

The blockade of T3 signaling specifically in brown adipocytes in adults has been achieved using the *Ucp1^CreERT2^* (Figure 1—figure supplement 1; Rosenwald et al., 2013). It was combined with two available floxed alleles to ascertain a full blockade of the T3 response in brown adipocytes. In the absence of tools to eliminate *Thra*, we used *Thra^AMI^*, a recombinant ‘floxed’ allele of the *Thra* gene, in which Cre-mediated recombination allows the expression of TRα1^L400R^, a dominant-negative version of the TRα1 receptor (Quignodon et al., 2007). TRα1^L400R^ prevents the recruitment of coactivators and is thus condemned to constitutively repress T3 target genes expression. Thus, we expect the effects of this knock-in to be stronger than a knock-out. Then, we used *Thrb^lox^,* a recombinant allele of *Thrb* gene, in which two loxP sequences flank the exon encoding the DNA-binding domain of TRβ1/TRβ2 (Winter et al., 2009). This approach eliminates T3 responsiveness. *Ucp1^CreERT2^xThra^AMI/+^Thrblox^lox/lox^* mice (Figure 1A) are called BATKO mice and compared with *Thra^AMI/+^Thrblox^lox/lox^* CTRL littermates.

**Figure 1—figure supplement 1.**
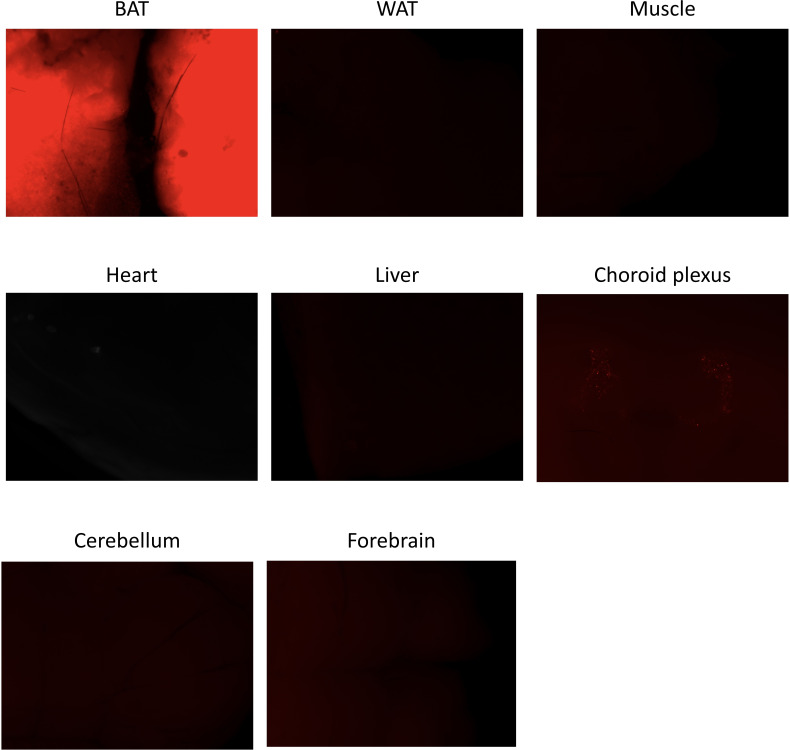
CRE activity in peripheral and central tissues. *Ucp1^CreERT2^:Rosa26^TdTomato^* reporter mice were dissected and their tissues observed under a Leica M205FA fluorescent stereomicroscope. Results are shown for one mouse.

**Figure 1—figure supplement 2.**
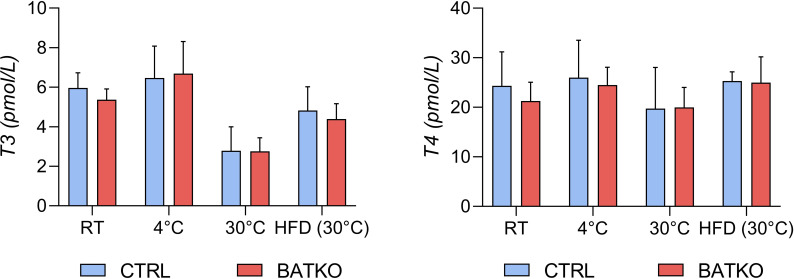
Serum concentration of TH in CTRL and BATKO mice. Serum concentration (in pmol/l) of free T3 (left panel) and free T4 (right panel) in CTRL and BATKO mice in different experimental conditions (RT: room temperature) (*n* = 4–5/group).

We verified that the *Thra^AMI^* allele was expressed in the BAT of BATKO mice (Figure 1B) and that *Thrb* expression was drastically reduced (93%) in the BAT of BATKO mice, but not in other tissues (Figure 1C). As expected, the T3-induced regulation of *Hr* expression, a classical T3 target gene in many tissues (Zekri et al., 2022), was selectively and almost completely lost in the BAT of BATKO mice (Figure 1D). The observed residual responses most likely reflect the presence in BAT of other cell types, like endothelial cells and immune cells (Biagi et al., 2021). Importantly, there was no significant difference between BATKO and CTRL mice in free T3 or free T4 serum levels, whatever the temperature and diet conditions used in this report (Figure 1—figure supplement 2). Altogether, BATKO mice present an altered T3 signaling specifically in brown adipocytes, allowing us to decipher the cell-autonomous functions of T3 in this cell type.

### Establishment of a catalog of TR direct target genes in BAT

We first aimed at establishing a complete list of TR direct target genes in brown adipocytes, defined as genes: (1) with a TR-binding site (TRBS) within 30 kb of the transcription start site (TSS) (Chatonnet et al., 2013), (2) which mRNA levels are rapidly (within 24 hr) increased in BAT in response to T3 and T4 (collectively TH), and (3) which induction is lost in BATKO mice, that is, controlled locally by TR in brown adipocytes (and thus, not secondary to the sympathetic stimulation of the tissue caused by the TH treatment).

**Figure 2.**
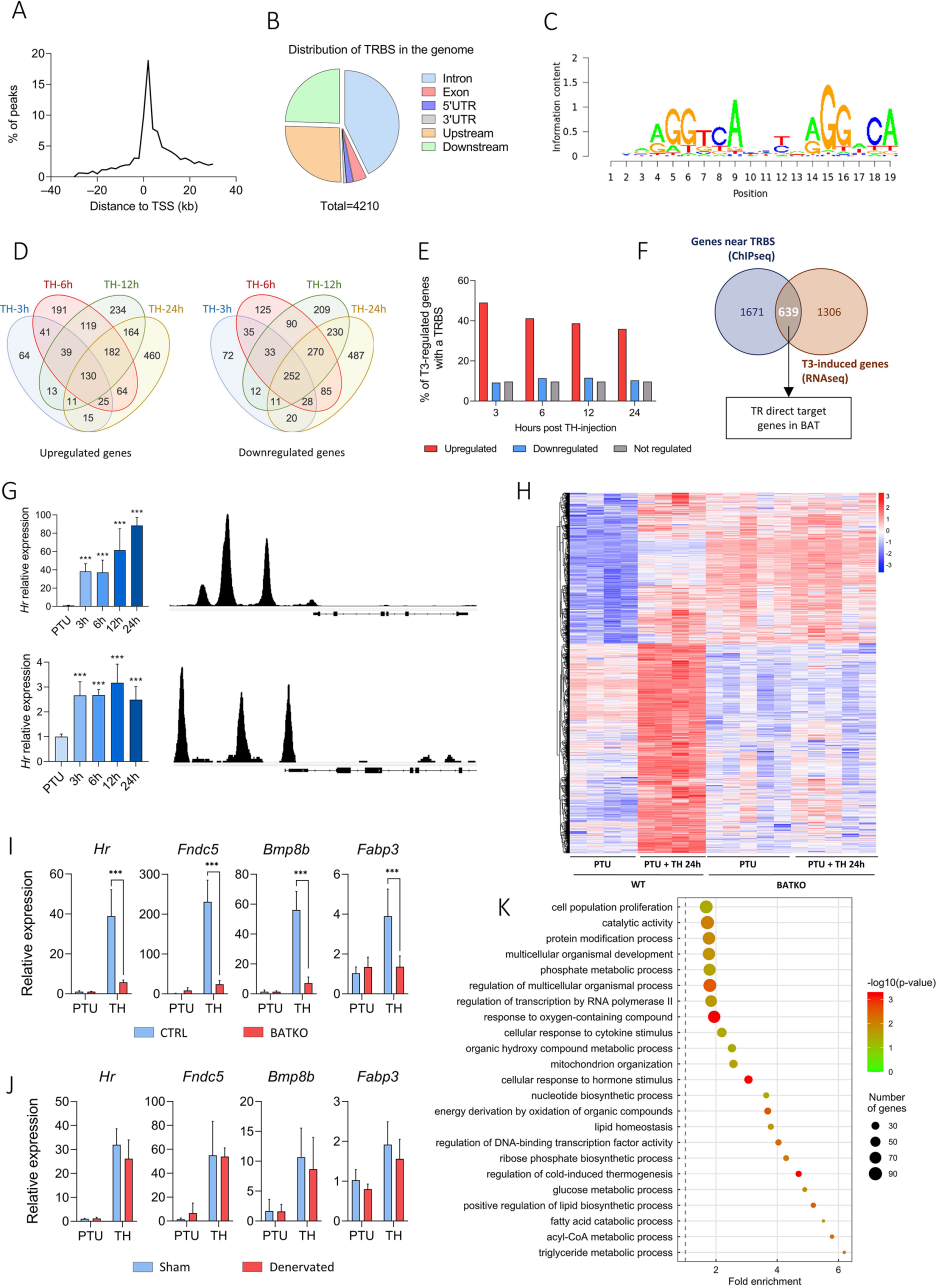
Identification of T3/TR target genes in brown adipocytes. (**A**) Consensus sequence found in brown adipose tissue (BAT) TRBS (thyroid hormone receptor-binding site), as identified by de novo motif search. (**B**) Frequency of TRBS distribution around transcription start sites (TSSs). (**C**) Pie chart of the 4210 TRBS distribution in the genome (UTR: untranslated region). (**D**) Venn diagrams of upregulated (left panel) and downregulated (right panel) genes after TH intraperitoneal injection in wild-type propylthiouracil (PTU)-treated mice for different periods. (**E**) Percentage of genes which possess a TRBS within 30 kb of their TSS among genes whose expression in the BAT is regulated or not by T3 (upregulated in red, downregulated in blue, not regulated in gray). (**F**) Venn diagram of genes whose expression is induced by T3 in at least one of the time points (in brown, RNAseq data) and genes with a TRBS within 30 kb of their TSS (in blue, chromatin immunoprecipitation sequencing [ChIPseq] data), that is, TR direct targer genes. (**G**) Left: Time-course analysis of *Hr* (top) and *Ucp1* (bottom) expression in BAT after 24 hr of TH treatment of wild-type hypothyroid mice (RT-qPCR). Statistical significance is shown for the different time points versus untreated PTU-fed mice (*n* = 4–6/group). Right: Extract of the TRBS in the *Mus musculus* genome browser around *Hr* (top) and *Ucp1* (bottom). (**H**) Heatmap representing in both CTRL and BATKO mice the expression of TR direct target genes upregulated after 24 hr of TH injection in CTRL hypothyroid mice. Colors represent the *z*-scores, see scale besides the heatmap (*n* = 4–5/group). Relative expression of several TR direct target genes 24 hr after TH treatment in (**I**) CTRL/BATKO and (**J**) Sham/denervated PTU-fed C57BL6/J mice. Statistical significance is shown for the comparisons between CTRL-TH and BATKO-TH, or SHAM-TH and BATKO-TH mice (*n* = 5–7/group). (**K**) Gene ontology dot plot of the 639 TR direct target genes. Only the ‘biological processes’ terms with a fold-enrichment >1.5 were kept. Some of the terms were shortened to increase readability without affecting the meaning. Error bars represent the standard deviation (SD). ***p < 0.001 for the indicated comparisons. Figure 2—source data 1.Raw data and compilation of T3/TR target genes in brown adipocytes. Figure 2—source data 2.Raw western blots for brown adipose tissue (BAT) denervation confirmation.Each lane represents a different sample, from sham mice (+) or denervated mice (−). Figure 2—source data 3.List of T3/TR target genes that also include PGC1α-binding sites.

*Ucp1^CreERT2^xThra^GS/+^* mice express specifically in brown adipocytes a GS-tagged version of TRα1 (Hirose et al., 2019; Richard et al., 2020) used for chromatin immunoprecipitation sequencing (ChIPseq). We identified 4210 TRBS, in the vicinity of 2311 genes, essentially located within 10 kb of the TSS (Figure 2A) and mostly in intronic sequences (Figure 2B). TRBS was preferentially present on motifs related to the so-called DR4 consensus sequence (AGGTCAnnnnRGGnCA), described as preferential for TR fixation (Flamant, 2016; Figure 2C).

**Figure 2—figure supplement 1.**
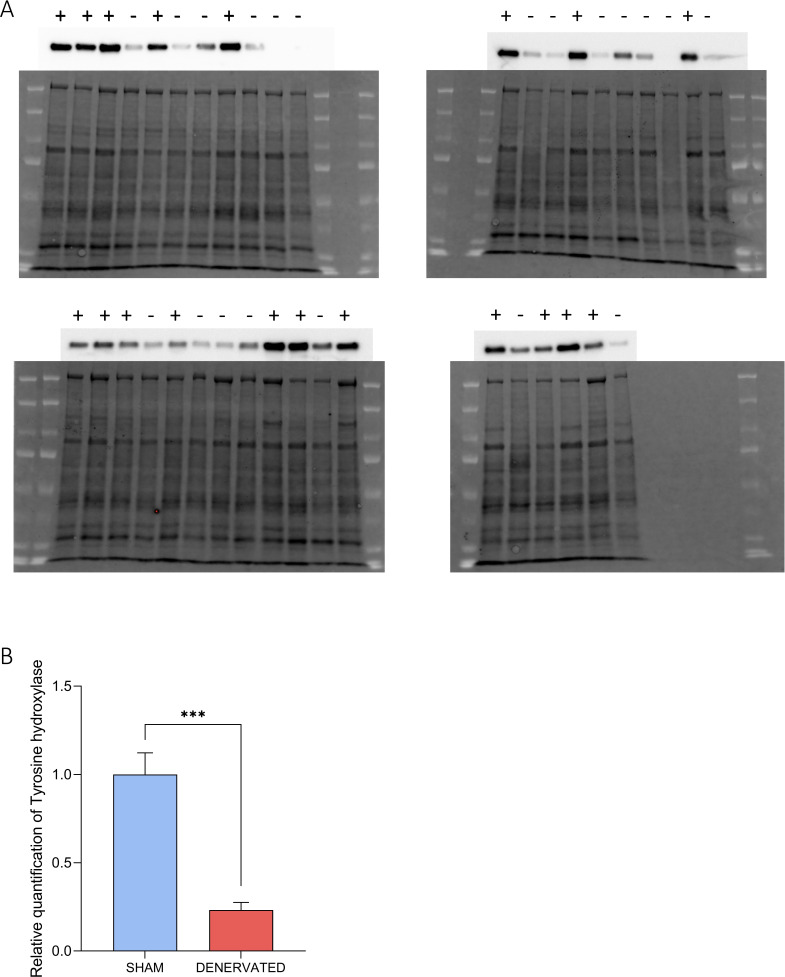
Confirmation of brown adipose tissue (BAT) denervation by quantification of tyrosine hydroxylase. (**A**) Western blots with total protein (bottom of the pictures) and tyrosine hydroxylase (top of the pictures) extracted from BAT. Each lane represents a different sample, from sham mice (+) or denervated mice (−). (**B**) Relative quantification of tyrosine hydroxylase protein in the BAT of sham and denervated mice. Quantity of tyrosine hydroxylase is relative to the quantity of total proteins. Error bars represent the standard error of the mean (SEM). ***p < 0.001 versus SHAM mice.

**Figure 2—figure supplement 2.**
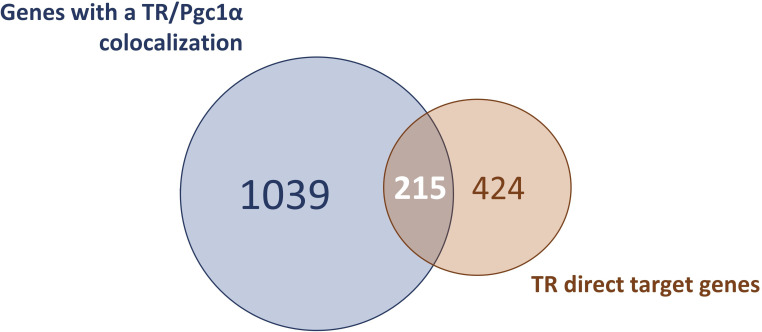
Comparison of TR direct target genes with the genes presenting a PGC1⍺-binding site. Venn diagram designed to highlight TR direct target genes that include a PGC1⍺-binding site.

Time-course analysis of BAT transcriptome in hypothyroid (PTU-fed) wild-type mice treated with TH for 3, 6, 12, or 24 hr was conducted by RNAseq. It revealed that a large number of genes were regulated (1946 upregulated, 1744 downregulated) in a time-dependent manner (Figure 2D). We observed that 49% of genes induced by T3 after 3 hr possess a TRBS within 30 kb of their TSS. This ratio fell below 10% for T3-repressed genes and for genes which expression was insensitive to T3 (Figure 2E). This suggests that downregulation of gene expression is not directly exerted by T3-bound TR, but is an indirect consequence of the TH treatment, either cell autonomous or resulting from sympathetic stimulation. Given these considerations, the rest of the study was restricted to positively regulated genes.

We then crossed the RNAseq and the ChIPseq datasets to obtain a curated list of 639 putative TR direct target genes (Figure 2F, Figure 2—source data 1), whose expression was most likely under the direct control of T3/TR signaling. As expected, this set of genes included two emblematic TR target genes: *Ucp1* (Martinez de Mena et al., 2010) and *Hr* (Engelhard and Christiano, 2004; Figure 2G). Heatmap representation showed that TR direct target genes previously found to be upregulated after 24 hr of TH treatment in wild-type mice were not responsive in BATKO mice (Figure 2H). Transcriptome results were confirmed by RT-qPCR for some of the identified TR direct targets (Figure 2I). We hypothesized that the residual response to T3 of BAT in BATKO mice might result either from the stimulation of brown adipocytes by the sympathetic system (López et al., 2010) or from the T3 response of BAT cells which do not express *Ucp1*, like endothelial or immune cells. To distinguish between these two possibilities, we chemically destroyed BAT noradrenergic terminals of wild-type PTU-fed mice (Figure 2—figure supplement 1, Figure 2—source data 2), treated or not afterwards with TH. T3-induced expression of target genes was mostly similar between sham and denervated mice (Figure 2J), indicating that, for the genes that we tested, the sympathetic stimulation was not involved.

According to gene ontology analysis, TR direct target genes were directly involved in the ‘regulation of cold-induced thermogenesis’ (Figure 2K), including *Ucp1* and *Ppargc1a*, two fundamental actors of this process. Interestingly, *Ppargc1a* encodes for PGC1⍺, a co-activator of TR (Yuan et al., 2013). Using a published list of PGC1⍺-binding sites (GSE110053) (Chang et al., 2018), we found that around 33% of TR direct target genes showed a co-localization of TR and PGC1⍺-binding sites (Figure 2—figure supplement 2), including genes involved in lipid metabolism as well as *Ucp1* and *Ucp3* (Figure 2—source data 3). In addition, many of TR direct target genes we identified were involved in mitochondrial transport, respiratory chain, catabolism, and biogenesis of lipids, as well as genes involved in the glycolysis and the citric acid cycle. Finally, we also found genes involved in proliferation, a process of BAT mid-term adaptation to physiological stressors like cold (Fukano et al., 2016). In summary, TR direct target genes belong to several biological processes, many of them being directly connected to BAT thermogenesis.

### Altered response of BATKO mice to temperatures below thermoneutrality

Based on the roles of TR target genes identified above, we predicted that BATKO mice would display alterations in BAT thermogenesis. At 23°C, neither body weight, nor body composition, nor metabolic rate were altered in BATKO mice, but food consumption was increased (Figure 3—figure supplement 1). As these small variations occurred at 23°C, which is a moderate cold exposure for mice (Reitman, 2018), we submitted them to a more drastic cold challenge.

**Figure 3.**
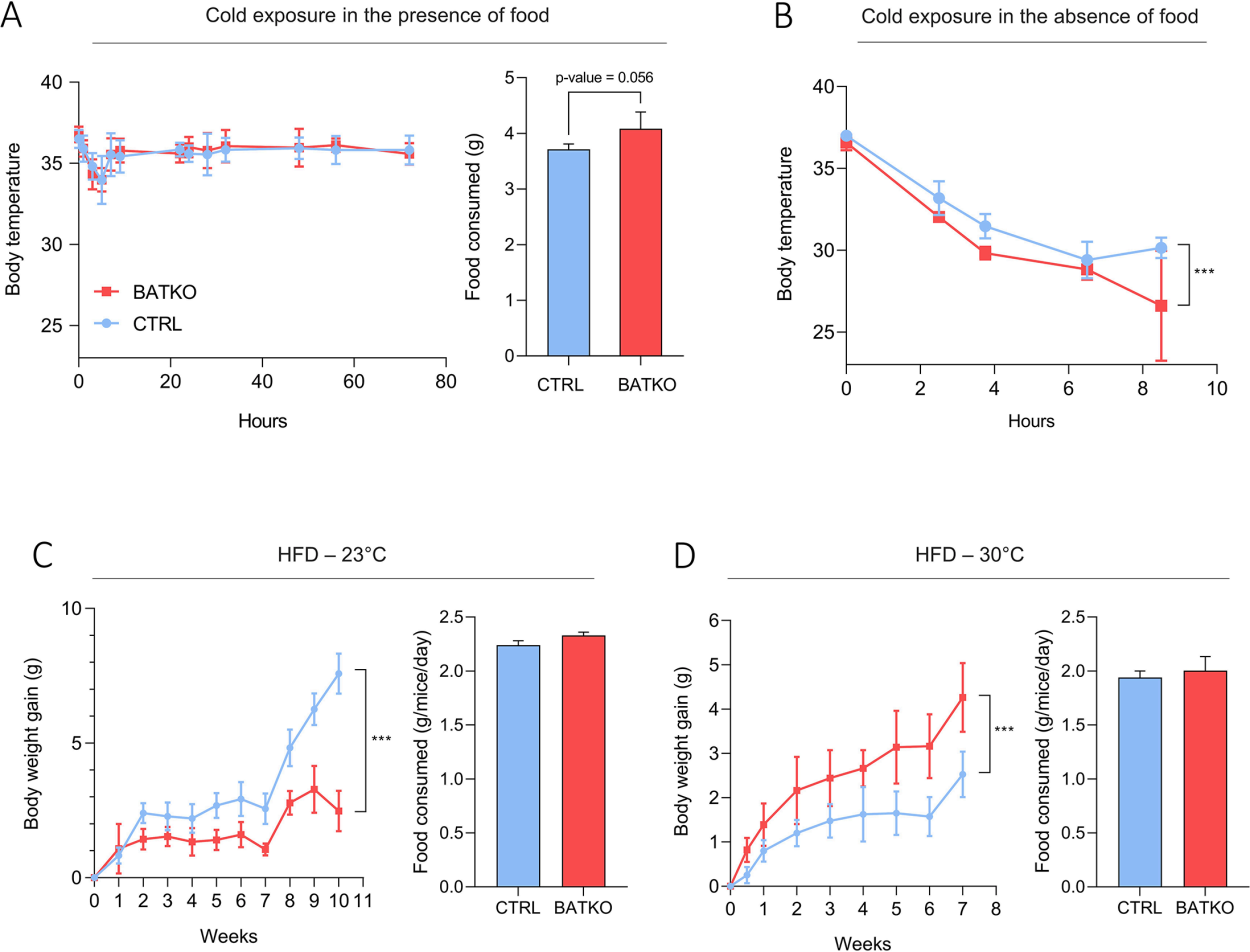
BATKO mice are cold sensitive. (**A**) Core body temperature (left) and food consumption (right, over 48 hr) of CTRL and BATKO mice exposed to 4°C in the presence of food (*n* = 5–7/group). BATKO mice tended to eat more than CTRL mice. (**B**) Core body temperature in CTRL and BATKO mice exposed to 4°C in the absence of food. After 8 hr of cold exposure, BATKO mice reached severe hypothermia and the experiment was stopped (*n* = 4–5/group). Body weight gain and food consumption of CTRL and BATKO mice at room temperature (**C**, *n* = 4–5/group) or 30°C (**D**, *n* = 5–8/group). Error bars represent the standard error of the mean. ***p < 0.001.

BATKO mice maintained their body temperature normally at 4°C during 72 hr but they tended again to consume more food than CTRL mice (Figure 3A). We thus combined cold exposure with fasting, causing a severe hypothermia in BATKO mice which led us to end the experiment (Figure 3B). A similar phenotype was obtained when TRβ was the only mutated TR, suggesting that a significant part of the effects mediated by T3 in BAT requires TRβ (Figure 3—figure supplement 2). Globally, alteration of T3 signaling in BAT requires a higher energy intake to maintain body temperature during a cold stress. This suggests that compensatory thermogenic processes are triggered to maintain body temperature in BATKO mice fed ad libitum. In that context, we notably observed that the browning of the WAT was exacerbated in BATKO mice (Figure 3—figure supplement 3).

**Figure 3—figure supplement 1.**
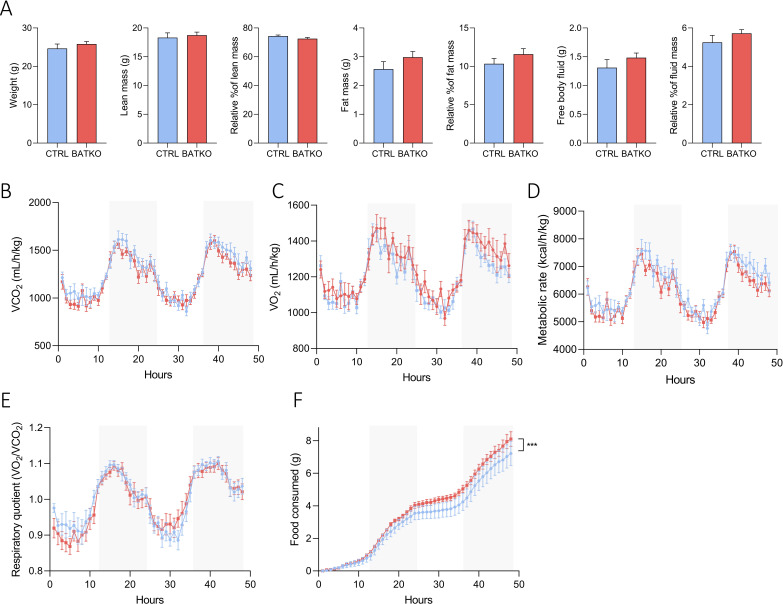
Body composition and indirect calorimetry in CTRL and BATKO mice. (**A**) Body composition of BATKO and CTRL mice measured by nuclear magnetic resonance (*n* = 7–9/group). (**B**) VCO_2_ (ml/hr/kg), (**C**) VO_2_ (ml/hr/kg), (**D**) metabolic rate, (**E**) respiratory quotient (ratio of VO_2_/VCO_2_), (**F**) cumulative food consumed by CTRL and BATKO mice, as monitored during 48 hr with 12 hr light/dark cyles (dark phases are represented by gray areas). Error bars represent the standard error of the mean (SEM). ***p<0.001.

**Figure 3—figure supplement 2.**
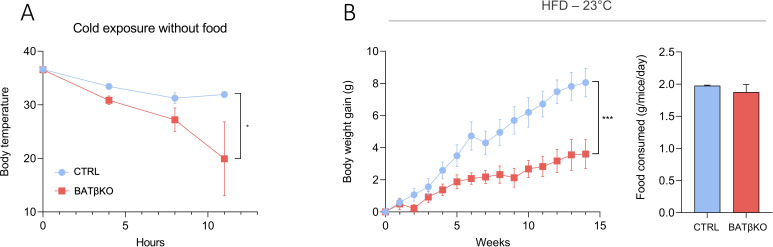
BATβKO mice are also cold sensitive. (**A**) Core body temperature in CTRL and BATβKO mice exposed to 4°C in the absence of food. After 11 hr of cold exposure, BATβKO mice reached severe hypothermia and the experiment was stopped (*n* = 6/group). (**B**) Body weight gain and food consumption of CTRL and BATβKO mice at room temperature (*n* = 4–6/group). Error bars represent the standard error of the mean. *p<0.05 and ***p < 0.001.

**Figure 3—figure supplement 3.**
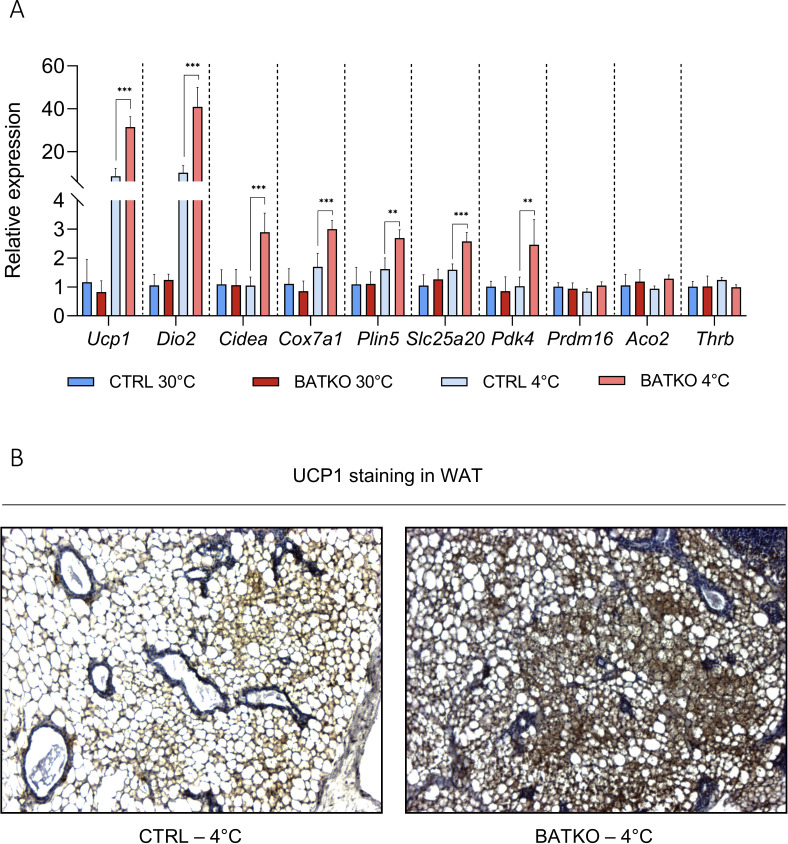
White adipose tissue (WAT) browning is exacerbated in BATKO mice. (**A**) Relative expression of genes in the WAT (*n* = 4–7/group) and (**B**) representative images of UCP1 staining in the WAT, in CTRL and BATKO mice after a 72 hr 4°C exposure (with ad libitum access to food). Browning was exacerbated in BATKO mice. *Thrb* was not affected in WAT. Statistical significance is shown for the CTRL 4°C versus BATKO 4°C group comparison. **p<0.01 and ***p<0.001 for the indicated comparisons.

HFD represents another challenge for the thermogenic capacity. BATKO mice gained less weight than CTRL mice during HFD at 23°C, despite similar food intake (Figure 3C). Again, this phenotype was reproduced when TRβ only was mutated, reinforcing the importance of TRβ in T3-mediated regulation of BAT thermogenic processes (Figure 3—figure supplement 2). Collectively, the resistance to diet-induced obesity suggests that mice with an altered T3 signaling in BAT have a higher energy expenditure. Higher energy expenditure was not observed in BATKO mice by 48 hr of indirect calorimetry (Figure 3—figure supplement 1) but even a minor difference could explain the subtle difference between BATKO and CTRL mice. HFD feeding was then conducted at thermoneutrality, eliminating the need to activate alternate mechanisms to defend body temperature. In this condition, the opposite result was observed, BATKO mice being more sensitive to diet-induced obesity than CTRL mice with similar food intake (Figure 3D). Collectively, these results point out that BATKO mice suffer from a reduced efficiency of BAT adaptive thermogenesis both in condition of cold exposure and excess of calories. When exposed to HFD at 23°C, the activation of alternative thermogenic processes combined with the defect in adipocytes thermogenesis results in a paradoxical resistance to obesity of BATKO mice.

### BAT TR signaling controls the expression of a subset of genes induced during cold exposure

**Figure 4.**
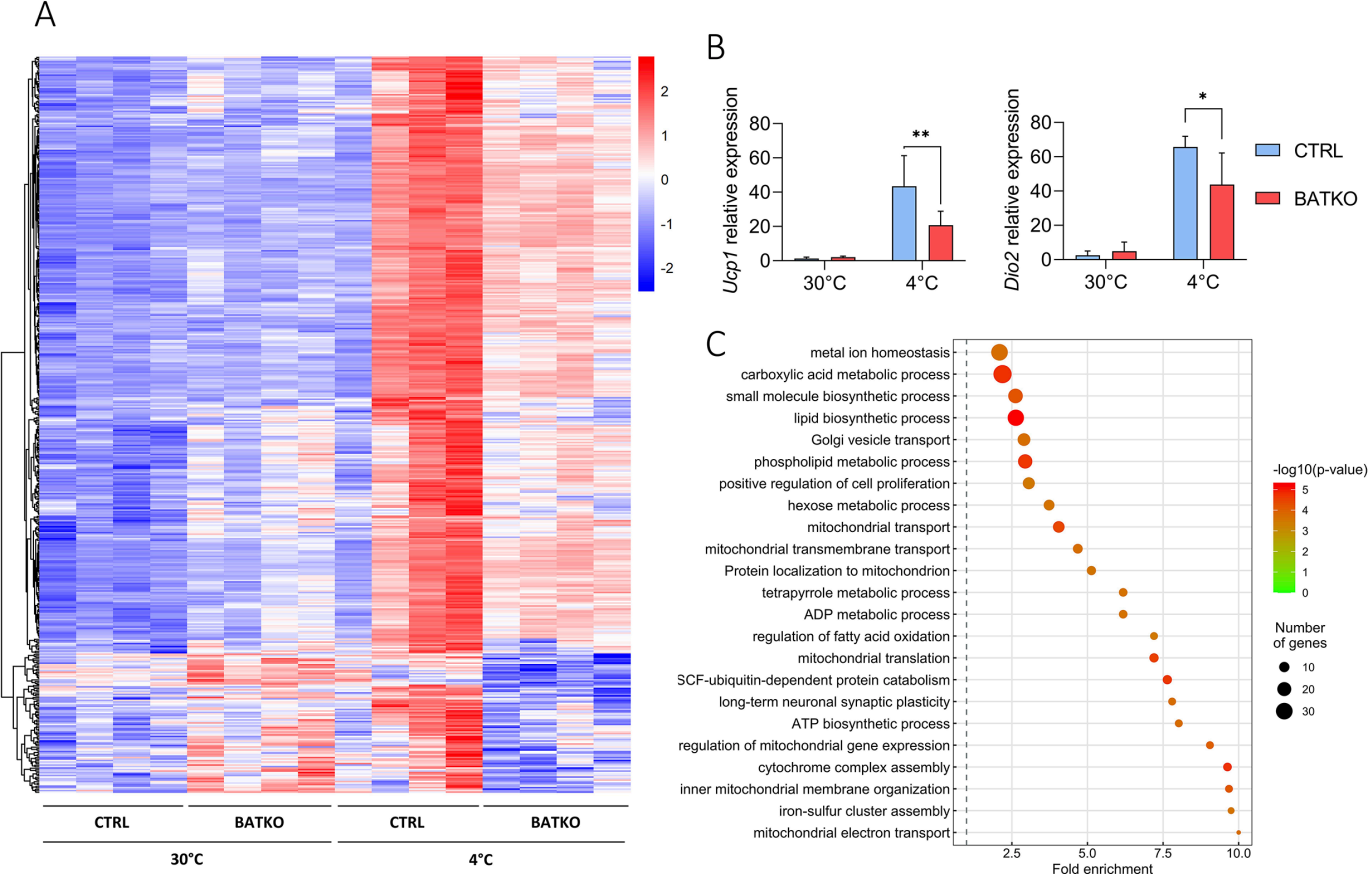
A subset of T3-regulated genes is activated during cold exposure and is necessary for an efficient brown adipose tissue (BAT) thermogenic response. (**A**) Heatmap representation of cold-responsive genes altered in BATKO mice, after 24 hr at 4°C. Colors represent the *z*-scores, see scale in heatmap (*n* = 3–5/group). (**B**) Relative mRNA expression of *Ucp1* (left panel) and *Dio2* (right panel) in CTRL and BATKO mice at 30°C or after 24 hr at 4°C. Statistical significance is shown for the comparison between CTRL and BATKO mice at 4°C (*n* = 4–6/group). (**C**) Gene ontology dot plot representation of biological processes enriched in the 491 genes inefficiently induced in BATKO mice at cold. Some of the terms were shortened to increase readability without affecting the meaning. Error bars represent the standard deviation (SD). *p < 0.05, **p < 0.01 for the indicated comparisons. Figure 4—source data 1.Transcriptome analysis of cold response in the brown adipose tissue (BAT) of both CTRL and BATKO mice. Figure 4—source data 2.List of T3/TR target genes which expression is affected in the brown adipose tissue (BAT) of BATKO mice during cold exposure.

To better understand the molecular response controlled by TR signaling in the BAT during cold response, we compared the BAT transcriptome of BATKO and CTRL mice after 24 hr at 4°C, in the presence of food. Among 2865 cold-induced genes (Figure 4—source data 1), 491 (17%) displayed a different response in BATKO mice (Figure 4A). For most of them, the cold induction was partially or completely lost in BATKO mice. Noteworthy, *Ppargc1a* was the only classical thermogenic marker that was part of this subset (Figure 4—source data 1). The highly stringent statistical interaction model that we used (Love et al., 2014) failed to reveal an influence of T3 on the cold response of the *Ucp1* and *Dio2* genes, two classical thermogenic markers. Thus, we used RT-qPCR to measure *Ucp1* and *Dio2* mRNA levels on a larger number of samples and found that the cold induction of these genes was indeed altered in BATKO mice (Figure 4B). Thus, the high stringency of the statistical model used avoids false positives, allowing to trustfully highlights genes of interest, but some of them can be missed due to a lack of statistical sensitivity.

Using gene ontology, we found that the genes which cold response was significantly altered in BATKO mice were often directly connected to thermogenesis (Figure 4C). This gene set notably included genes involved in mitochondrial activity and respiratory chain. Other genes were involved in glycolysis*,* Krebs cycle, lactate metabolism, and glucose transport which have all been shown to be crucial to fuel BAT thermogenesis (Jeong et al., 2018). Genes involved in both lipolysis/fatty acid oxidation and lipogenesis, two processes required for an appropriate lipid use during thermogenesis, were also altered. Finally, a gene set reflecting cell proliferation was activated (Figure 4—source data 2). Restriction of this list to the TR direct target genes (Table 1) also highlighted secreted peptides (*Bmp8b* and *Fgf21*) and enzymes participating in heat-producing creatine futile cycle (*Alpl*). *Ppargc1a* still belonged to this list of restricted genes of interest, reinforcing its importance in TR-mediated regulation, as abovementioned. Collectively, the BAT transcriptome of BATKO mice evidenced several altered pathways crucial for BAT thermogenesis that could explain their phenotype.

**Table 1.** List of T3/TR target genes dysregulated during cold exposure in BATKO mice.

Biological function	TR-dependent cold-induced genes
**Lipid metabolism**	*Fabp3*, *Pla2g12a*, *Acsl5*, *Aspg*, *Mcee*
**Glucose metabolism**	*Slc2a4*, *Ogdh*, *Idh3a*
**Cell cycle progression**	*Cux1*, *Ccnd1*
**Mitochondrial activity**	*Ppargc1α*, *Coq10a*, *Uqcc2*, *Cyb561*
**Futile cycle**	*Alpl*
**Secreted peptides**	*Bmp8b*, *Fgf1*

### T3 in BAT controls BAT proliferation

RNAseq analysis revealed that TH might participate in BAT proliferation, both during hyperthyroidism and cold exposure. After 5 days of TH treatment, wild-type PTU-fed mice displayed an overexpression of 72% of a group of genes previously described to be involved in cell cycle (Whitfield et al., 2002; Figure 5—figure supplement 1). This translated into an effective increase in cell proliferation when TH treatment was associated with an injection of EdU 24 hr before sampling. This effect was insensitive to BAT denervation (Figure 5—figure supplement 1), suggesting that it most likely results from a cell-autonomous response to T3.

**Figure 5.**
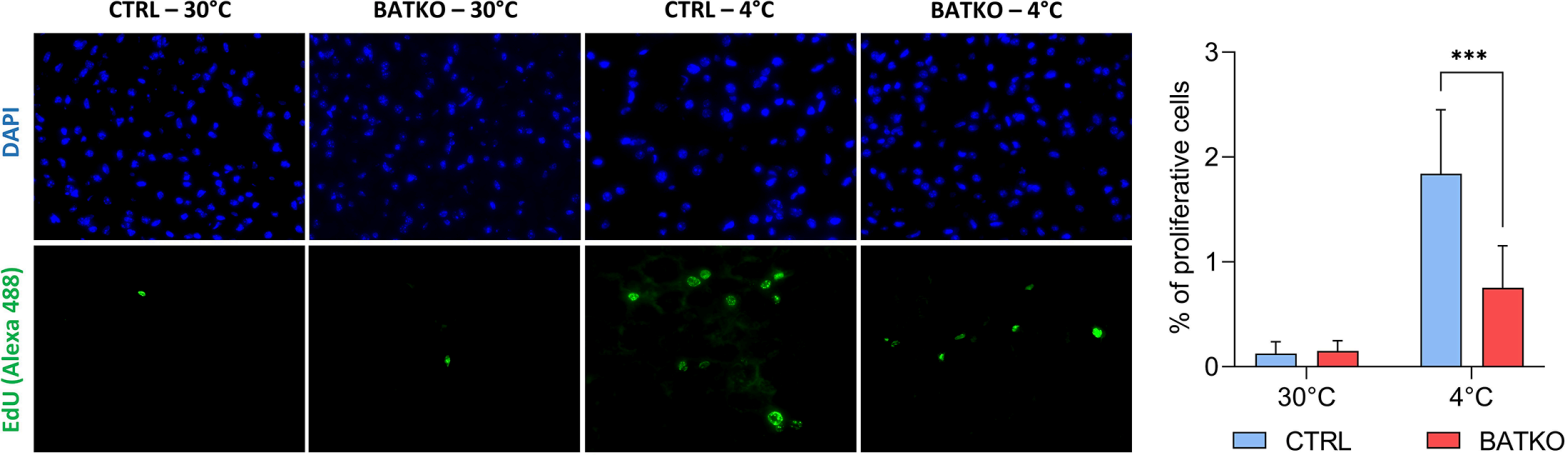
Local control of brown adipocytes proliferation by T3. Representative images (left) and quantification (right) of EdU-positive proliferative cells in brown adipose tissue (BAT) from both CTRL and BATKO mice exposed 72 hr to 4°C and injected with EdU after 24 and 48 hr of cold. Nuclei are stained in blue with DAPI. Percentage of proliferative cells is the ratio of proliferative cells on the number of nuclei (*n* = 4–7/group). Statistical significance is shown for the comparison CTRL 4°C versus BATKO 4°C. Error bars represent the standard deviation (SD). ***p < 0.001r for the indicated comparisons.

As predicted by the transcriptomic data after 24 hr of cold exposure, we also observed that proliferation is triggered in the BAT when CTRL mice were exposed 72 hr to cold, but 2.5-fold less proliferative cells were observed in BATKO mice, in the same conditions (Figure 5).

**Figure 5—figure supplement 1.**
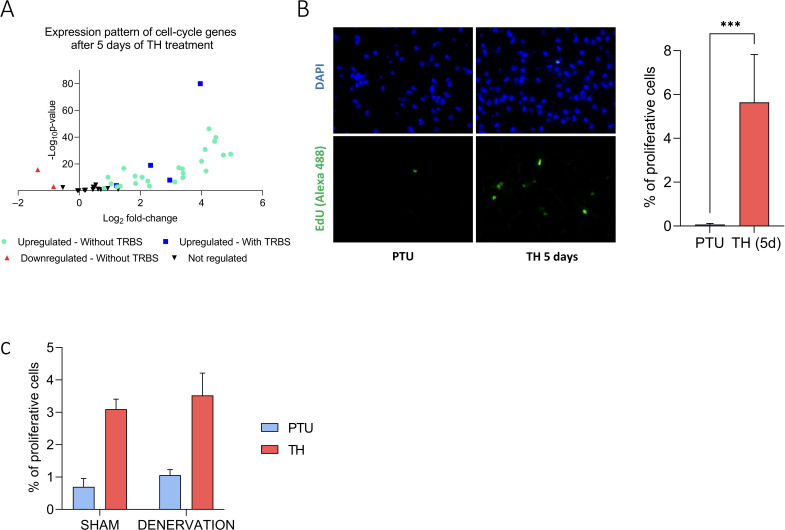
TH trigger brown adipose tissue (BAT) proliferation. (**A**) RNAseq expression pattern of cell cycle genes (Whitfield et al., 2002) after 5 days of TH treatment in wild-type propylthiouracil (PTU)-fed mice. Genes are divided into four groups, according to their regulation by TH treatment (*n* = 4/group) and the presence of a thyroid hormone receptor-binding site (TRBS) within 30 kb from their promoter. (**B**) Representative images (left) and quantification (right) of EdU-positive proliferative cells (green cells) in BAT from wild-type hypothyroid mice treated or not with TH during 5 days and co-injected with EdU at day 4. Nuclei are stained in blue with DAPI. Percentage of proliferative cells is the ratio of proliferative cells on the number of nuclei (*n* = 5–6/group). (**C**) Quantification of EdU-positive cells in BAT from PTU-fed denervated or sham mice treated or not with TH during 5 days and co-injected with EdU at day 4. Percentage of proliferative cells is the ratio of proliferative cells on the number of nuclei (*n* = 5/group). Error bars represent the standard deviation (SD). ***p<0.001.

As most of the cell-cycle genes induced by T3 do not have a TRBS within 30 kb (Figure 5—figure supplement 1), the link between the TR direct target genes and proliferation in BAT yet remains uncertain. *Ccnd1*, encoding cyclin D1, is one of the TR direct target gene (Figure 2—source data 1) and also belongs to the genes that are under the control of TH signaling during cold exposure (Figure 4—source data 2). It is thus an interesting candidate to play a significant part in this process.

## Discussion

T3 has been for a long time under the lights of metabolism research for its ability to regulate energy expenditure in many tissues, including the BAT. However, neither the exhaustive list of TR direct target genes in BAT, nor the contribution of BAT in T3-mediated regulation of energy expenditure have been elucidated so far. Here, we present an unprecedented in-depth analysis of T3 direct influence on gene expression in brown adipocytes and developed a transgenic mice model with a brown-adipocyte-specific suppression of T3 signaling. To our knowledge, this model is currently the only one allowing a suppression of T3 signaling specifically in brown adipocytes at adult stages. This is a significant step forward as it allows (1) to dissect the contribution of BAT in the T3-mediated increased in energy expenditure, (2) to distinguish the hypothalamic (López et al., 2010) versus local control of BAT thermogenesis, and (3) to bypass any developmental alteration mediated by the early lack of T3 signaling (Hall et al., 2010). Collectively, this new model showed that T3 signaling was pivotal for BAT adaptive thermogenesis, regulating the management of thermogenic fuels as well as the plasticity of the tissue.

Combined with transcriptome analyses, the genome-wide study of TRα1 chromatin binding allowed us to identify 639 genes whose transcription is most likely controlled by liganded TR in brown adipocytes. This set of genes only partially overlaps with the ones described in other cell types, probably as a result of differential chromatin occupancy (Chatonnet et al., 2013; Hirose et al., 2019; Richard et al., 2020) and the presence of cell-specific transcription cofactors. One of these cell-specific cofactors is PGC1α (encoded by *Ppargc1a*) which we found here to be a TR direct target gene. As PGC1α is a transcriptional coactivator of TR (Yuan et al., 2013), it confirms previous hypothesis that TR and PGC1α are involved in an auto-regulatory feed-forward loop (Wulf et al., 2008). In line with this, we found that 33% of the TRBS in brown adipocytes are also occupied by PGC1α, notably for genes involved in lipid metabolism or directly involved in thermogenesis, like *Ucp1* and *Ucp3* (Cannon and Nedergaard, 2004; Silvestri et al., 2020). PGC1α is thus likely to play a pivotal role in the T3-dependent regulation of energy metabolism in brown adipocytes. As PGC1α is also a coactivator of several other nuclear receptors, notably PPARγ and ERRα, its overexpression might generate a cross-talk with other signaling pathways.

One of the main challenges of our study was to make a distinction between a local influence of T3 and an indirect consequence, resulting from the hypothalamic response to T3 (López et al., 2010). The BAT response to T3 is largely lost in BATKO mice and conserved in denervated mice, suggesting that the changes in gene expression observed in BAT mainly reflect the cell-autonomous response initiated by TR in brown adipocytes and not the effect of T3 mediated by the hypothalamus (López et al., 2010). Residual responses to T3 in BATKO may reflect the sensitivity of other cells types present in the BAT which do not express the *Ucp1^CreERT2^* transgene or an incomplete effect mediated by tamoxifen. Otherwise, they could be secondary to a modification of circulating peptides caused by the T3 treatment on other organs. For instance, *Fgf21* expression is induced by T3 in the liver (Adams et al., 2010) and the resulting secreted peptide has been shown to promote BAT thermogenesis (Sáenz de Urturi et al., 2022). We found indication for a reciprocal influence as several TR direct target genes in brown adipocytes encode secreted factors and adipokines (*Apln*, *Fdcn5*, and *Bmp8b*), that are able to act in other organs. This suggests that T3 can influence a complex network of cross-talks in the organism.

T3 signaling in BAT is triggered in CTRL mice during cold exposure as it was previously shown by the activation of type 2 deiodinase activity in similar conditions (de Jesus et al., 2001). This activation of T3 signaling represents a significant fraction of the cold response, since 17% of the gene regulations induced in the BAT by cold exposure in CTRL mice are lost in BATKO mice. We showed that T3 signaling in the BAT controls the expression of genes crucial for both lactate metabolism (*Ldha*) or glycolysis (*Hk1*, *Aldoa*, *Pkm*, and *Pfkl*), two pathways essential for BAT thermogenesis (Jeong et al., 2018). T3 also controls genes involved in lipid metabolism, which is also congruent with previous observations revealing that T3 signaling is required for efficient lipogenesis (Christoffolete et al., 2004) and lipolysis (Oppenheimer et al., 1991). Here, we bring a broader picture by identifying all the genes which expression is regulated by T3 for these processes.

Interestingly, we showed that *Ppargc1a* was among impacted genes. As it coactivates other nuclear receptors, we can imagine that part of the genes dysregulated in BATKO mice can be attributed to other signaling pathways downstream of PGC1α. On the other hand, we observed that other classical thermogenic markers were not the most obviously affected in BATKO mice. Indeed, *Ucp1* and *Dio2* could not be detected as differentially expressed by our stringent whole-transcriptome approach but only by targeted expression assessment. Among TR-dependent cold-induced genes, we rather detected *Alpl*, a phosphatase required in brown adipocytes for UCP1-independent thermogenesis derived from futile creatine cycle (Sun et al., 2021). Thus, T3 signaling in BAT coordinates the expression of genes involved in both UCP1-dependent and UCP1-independent thermogenesis. Finally, we showed that T3 signaling in brown adipocytes controls cell proliferation both during cold exposure and upon hyperthyroidism. This concurs with a recent study showing that T3 promotes BAT proliferation in adult mice in a cell-autonomous manner (Liu et al., 2022). Also, the link between TR target genes and the cell cycle remains uncertain. The direct activation of *Ccnd1*, encoding cyclin D1, provides a hypothetical link between T3 and proliferation. This intertwining might also occurs through the regulation of cell cycle gene regulators, as already demonstrated for CREB and AP-1 (Pascual and Aranda, 2013).

Thus, inefficient handling of metabolic fuels, reduction of the tissue plasticity, and defect in thermogenic biochemical pathways can explain the apparent default in BAT thermogenesis. This can be compensated by an increased energy consumption which could explain why BATKO mice gained less weight than CTRL mice at room temperature, which represents a mild cold challenge (Škop et al., 2020). This extra-energy consumption might favor the onset of other thermogenic mechanisms, as observed with the exacerbated WAT browning. However, WAT browning’s ability to consume a significant amount of energy has recently been called into question (Challa et al., 2020). Although we failed to measure a striking difference in BATKO mouse oxygen consumption at room temperature over a 48-hr period, a minor increase in the respiratory quotient might have long-term consequences. At thermoneutrality, where no compensatory mechanisms are required to maintain body temperature, BATKO mice became hypersensitive to diet-induced obesity. This reflected the alteration of diet-induced BAT thermogenesis and the inability of this tissue to optimally use metabolic substrates and thus expend energy. A similar phenotype was previously observed for other mouse models deficient for BAT thermogenesis (Castillo et al., 2011).

In humans, BAT has triggered a lot of interest for its ability to increase energy expenditure, which could be used in the fight against obesity. Interestingly, T3 levels also correlate with BAT activity in humans (Bredella et al., 2012; Lahesmaa et al., 2014). Hyperthyroid patients have higher glucose uptake in BAT, and this effect is neutralized after treatment to restore the euthyroid state (Lahesmaa et al., 2014). Therefore, T3 also plays a role in human BAT thermogenesis. Whether the mechanisms regulated by T3 in human BAT are the same as described here in mice is an open question. While T3 cannot be used per se to increase energy expenditure in humans due to cardiac side effects (Biondi et al., 1993), its hybridization to other molecules could allow to target it selectively to BAT, as already done for liver (Finan et al., 2016).

In conclusion, we used a combination of both omics data and BAT-specific TR-signaling alteration in mice to introduce an unprecedented view of T3 cell-autonomous response in brown adipocytes. We showed that T3 signaling in BAT controls both key metabolic pathways and tissue plasticity that are essential for adaptive thermogenesis. These results represent a valuable database that pave the way for further metabolic studies to detail the molecular implications of T3 signaling during BAT adaptive thermogenesis. Finally, as T3 acts in many other tissues, a similar approach could be used to put together the puzzle of T3 influence in energy expenditure.

### Limitations of the study

One of the objectives of this study was to define a catalog of TR direct target genes in brown adipocytes, based on a combination of RNAseq and ChIPseq. Both TRα and TRβ are present in the BAT (Minakhina et al., 2020) but we only performed the TRα1 ChIPseq due to the absence of adequate tools for the TRβ ChIPseq. The cistromes of the two receptors might not fully overlap, and we might have missed a fraction of the TRBS on the genes of interest. However, this limitation should be tempered since previous data have indicated that TRβ-selective binding site are infrequent in the mouse genome (Chatonnet et al., 2013). Another limitation is that we assigned genes to a given TRBS by considering only its distance to the transcription starting site. Chromatin 3D organization and insulation are well known to influence the interactions between distant regulatory elements, and the linear distance is only partially informative. The additional genomic investigations required to overcome these limitations remain for the moment hardly feasible in mouse tissues. Finally, we did not look at protein changes while post-translational events could occur.

## Materials and methods

**Key resources table:** 

Reagent type (species) or resource	Designation	Source or reference	Identifiers	Additional information
Strain, strain background (male mice)	C57BL/6J (male)	Charles River		
Genetic reagent (male mice)	C57BL/6J-BATKO (male)	This paper		See ‘Material and methods’, section ‘Genetically modified mouse models’
Genetic reagent (male mice)	C57BL/6J-BATβKO (male)	This paper		See ‘Material and methods’, section ‘Genetically modified mouse models’
Antibody	Anti-UCP1 (rabbit polyclonal)	Abcam	Abcam: ab10983	1:400
Antibody	Anti-rabbit linked to peroxidase (goat polyclonal)	Promega	Promega: W401B	1:300
Antibody	Anti-tyrosine hydroxylase (rabbit polyclonal)	Merck	Merck: AB152	1:500
Antibody	Anti-rabbit IgG linked to peroxidase (goat polyclonal)	Bio-Rad	Bio-Rad: STAR124P	1:5000
Sequence-based reagent	See Table 2	This paper		
Commercial assay or kit	Pierce BCA Protein Assay Kit	Thermo Fisher Scientific	Thermo Fisher Scientific: 23225	
Commercial assay or kit	Clarity Western ECL Substrat	Bio-Rad	Bio-Rad: 1705060	
Commercial assay or kit	Diaminobenzidine staining	Sigma-Aldrich	Sigma-Aldrich: D5905	
Commercial assay or kit	Click-iT EdU Cell Proliferation Kit	Thermo Fisher	Thermo Fisher: C10337	
Commercial assay or kit	RNA SENSE Kit	Lexogen		
Commercial assay or kit	Accel-NGS 2S Plus DNA kits	Swift Biosciences		
Chemical compound, drug	Tamoxifen	Sigma-Aldrich	Sigma-Aldrich: T5648	
Chemical compound, drug	PTU-containing diet	Harlan Teklad	Teklad Custome Diet: TD95125	
Chemical compound, drug	T3	Sigma-Aldrich	Sigma-Aldrich: T2877	
Chemical compound, drug	T4	Sigma-Aldrich	Sigma-Aldrich: T2376	
Chemical compound, drug	6-OHDA	Sigma-Aldrich	Sigma-Aldrich: H4381	
Other	Cobas 600	Roche		T3/T4 serum quantification
Other	Leica TP1020	Leica		Tissue section dehydration

**Table 2.** Primer sequences.

Gene	Primer forward	Primer reverse
*Ucp1*	AAGCTGTGCGATGTCCATGT	AAGCCACAAACCCTTTGAAAA
*Hr*	AGAGGTCCAAGGAGCATCAAGG	TTCCTCTTGTTGCTCTGCCTCC
*Fndc5*	ATGAAGGAGATGGGGAGGAA	GCGGCAGAAGAGAGCTATAACA
*Bmp8b*	ACCTGTACCGTGCCATGACG	CGGTCGCGTTCCACTATGTTG
*Idh3a*	AAGAGGTTTTGCTGGTGGTGTTC	TTGCGCTCCTCCCACTGAATAG
*Fabp3*	CATCGAGAAGAACGGGGATA	TCATCTGCTGTCACCTCGTC
*Dio2*	AACAGCTTCCTCCTAGATGCC	ATTCAGGATTGGAGACGTGC
*Cidea*	GGACAGAAATGGACACCGGGTAG	TGACATTGAGACAGCCGAGGAAG
*Cox7a1*	AGGCTCTGGTCCGGTCTTTTAG	GGTCATTGTCGGCCTGGAAG
*Plin5*	GCGCCACACAGCAGAATGTC	GGCAAAGCCACCACTCGATTC
*Slc25a20*	TCAGGCTTCTTCAGGGGAGAAC	CCACTGGCAGGAACATCTCG
*Pdk4*	TTTCCAGGCCAACCAATCCAC	GTGGCCCTCATGGCATTCTTG
*Prdm16*	TAGCTGCTTCTGGGCTCAAGG	ACGTCACCGTCACTTTTGGC
*Aco2*	TGCCTAAGGTGGCTGTACCATC	CACTTCCTGGTTTATGTCCTTGGC
*Thrb*	CTCTTCTCACGGTTCTCCTC	AACCAGTGCCAGGAATGT

### Animal procedures

All experiments were carried out in accordance with the European Community Council Directive of September 22, 2010 (2010/63/EU) regarding the protection of animals used for experimental and other scientific purposes. The research project was approved by a local animal care and use committee (C2EA015) and authorized by the French Ministry of Research.

### Genetically modified mouse models

The genetic background of all mice that were used in the present study was C57BL6/J. *Thra^AMI/+^* (Quignodon et al., 2007), *Thrb^lox/lox^* (Winter et al., 2009), and *Ucp1^CreERT2^* (Rosenwald et al., 2013) mouse lines were crossbred to introduce the different recombinant alleles in *Ucp1^CreERT2^xThra^AMI/+^Thrblox^lox/lox^* mice. The expression of the *Thra^AMI^* allele allows the expression of the TRα1^L400R^ mutant, which has dominant-negative properties over TRα and TRβ, after Cre/loxP-mediated excision of a stop cassette (Quignodon et al., 2007). Despite the persistence of one intact *Thra* allele in *Thra*^AMI/+^ mice after Cre-recombinase action, the dominant-negative action of TRα1^L400R^ eliminates the capacity of cells to respond to T3. *Thrb^lox^* has 2 tandem-arranged loxP sequences, allowing Cre-mediated excision of exon 3 of the *Thrb* gene, which encodes the DNA-binding domain of the TRβ1/TRβ2 receptor, resulting in a frameshift and a loss of function (Winter et al., 2009). In the present study, we used mice of the *Ucp1^CreERT2^* line, in which CreERT2 expression is under the control of the *Ucp1* promoter (Rosenwald et al., 2013). In the absence of physiological stressors, *Ucp1* is specifically expressed in brown adipocytes, thus restricting Cre-mediated recombination to this cell type. CreERT2-recombinase action occurs after its translocation to the nucleus, allowed by tamoxifen treatment. Tamoxifen was injected intraperitoneally every day during 5 days at 50 mg/kg of mice.

In *Ucp1^CreERT2^* mice, we used a *Rosa26^TdTomato^* reporter transgene, also known as Ai9 (Figure 1—figure supplement 1; Madisen et al., 2010), we verified the absence of recombination activity outside BAT, except for a small fraction of cells present in the choroid plexus. In the following, mice with the *Ucp1^CreERT2^xThra^AMI/+^Thrblox^lox/lox^* genotype were called BATKO. *Thra^AMI/+^Thrblox^lox/lox^* littermates, also injected with tamoxifen, were used as controls (CTRL). To explore the specific of TRβ for the observed phenotypes in BATKO mice, we also used *Ucp1^CreERT2^xThrb^lox/lox^* called BATβKO mice. *Thrb^lox/lox^*, also injected with tamoxifen, were used as controls.

In order to address chromatin occupancy by TR specifically in brown adipocytes, we used *Thra^GS/+^* mice (Hirose et al., 2019) to generate ad hoc Ucp1^CreERT2^xThra^GS/+^ transgenic mice. These mice were generated by knocking in the *Thra* locus a sequence encoding TRα1 fused with protein G and streptavidin protein (GS) after a floxed stop cassette. In the presence of the *Ucp1^CreERT2^*, tamoxifen injection allows the recombination at the loxP sites that excise the stop cassette specifically in brown adipocytes, allowing the expression of the GS-TRα1 only in this cell type. This strategy has already been successfully used in other tissues, including striatum and heart (Richard et al., 2020; Hirose et al., 2019).

### Experimental animal procedures

We used 2- to 5-month-old male mice for experiments. Genetically modified mice were generated in our own animal facility, whereas wild-type C57BL6/J mice were ordered from a commercial supplier (Charles River). Mice were fed ad libitum with LASQC Rod16 R diet (Altromin, Germany) and housed under recommended conditions (notably, at room temperature, i.e., 23°C). Hypothyroidism in adult animals was induced as previously described, with 14 days of treatment with a propylthiouracil (PTU)-containing diet (Harlan Teklad TD95125, Madison, WI) (Weiss et al., 1998). It was combined in some cases by hyperthyroidism induced by intraperitoneal injections of a T3/T4 mix (T4 at 2 μg/g of mice and T3 at 0.2 μg/g of mice, Sigma-Aldrich), daily for the five last days or once at day fourteen of the PTU treatment. T3, the active compound, was not injected alone to get close to hyperthyroid conditions met in vivo.

Before assessment of cold response, mice were housed for 10 days at 30°C (with normal 12 hr light/dark cycles) and subcutaneously implanted with IPTT-300 transponders (Plexx BV, Netherlands). The mice were then housed in pairs without enrichment and placed at 4°C during the indicated period. Infrared thermography was performed on awake mice after 48 hr of cold exposure, using an infrared camera (FLiR Systems, Inc). For cell proliferation assessment, EdU (Thermo Fisher) was intraperitoneally injected twice (100 mg/kg, after 24 and 48 hr of cold exposure) and mice were killed after 72 hr of cold exposure. This allowed to stain proliferative cells over the last 48 hr of cold exposure.

At the end of experiments, mice were anesthetized by an intraperitoneal injection of xylazine (25 mg/kg) and ketamine (130 mg/kg) mixture. Blood was drawn from the vena cava and collected in heparin-coated tubes to retrieve plasma. Several tissues and organs were dissected and either directly processed for histology or snap frozen and stored at −80°C for later RNA preparation.

### Indirect calorimetry and body composition measurement

Body composition was measured in awake mice by low-field nuclear magnetic resonance with a Minispec LF90II device (Bruker). Phenomaster metabolic cages were used for indirect calorimetry measurements (TSE Systems, Berlin, Germany). Mice were first placed in individual cages for a 24-hr period of habituation to isolation, after which oxygen consumption (VO_2_) and carbon dioxide rejection (VCO_2_) were continuously recorded for 48 hr, under a normal 12 hr light/dark cycle. VO_2_ and VCO_2_ were expressed as ml/hr/kg of mice. Respiratory quotient was obtained as the VO_2_ / VCO_2_ ratio. The metabolic rate was calculated according to the Weir formula (Weir, 1990) as following: Metabolic rate (kcal/min) = 3.94*VO_2_ + 1.1*VCO_2_.

### BAT denervation

Chemical denervation of BAT sympathetic nerve endings was performed in wild-type PTU-fed mice under isoflurane 2% anesthesia and ketoprofen (1 mg/kg) analgesia. This technique, which involves injections of 6-hydroxydopamine (6-OHDA), a neurotoxin selective for sympathetic neurons, permits a specific denervation of sympathetic neurons, while keeping the sensory fibers intact (Nitta et al., 1992). The effects of sympathetic denervation were compared to those obtained after vehicle injection (0.15 mol/l NaCl and 1% ascorbic acid, sham mice). Briefly, the two lobes of interscapular BAT were exposed through a midline skin incision along the upper dorsal surface and gently separated from the skin with surgical forceps. Then, injections of 6-OHDA (Sigma-Aldrich) were performed directly into each lobe of the interscapular BAT. For each lobe, 10 µl (10 mg/ml) was injected in several times (10 injections of 1 µl) using a Hamilton syringe (i.e., 20 µl/mice). The skin incision was then closed with several surgical stitches. Animals were allowed to recover for 5 days before further experiments.

### Western-blot analysis

Cell extracts from BAT were lysed in standard lysis buffer (20 mM Tris–HCl, pH 8, 138 mM NaCl, 1% NP40, 2.7 mM KCl, 1 mM MgCl_2_, 5% glycerol, 5 mM EDTA (ethylenediaminetetra-acetic acid), 1 mM Na_3_VO_4_, 20 mM NaF, 1 mM dithiothreitol, 1% protease inhibitors), and homogenized using FastPrep (MP Biomedicals). Proteins were assayed in triplicate with Pierce BCA Protein Assay Kit (Thermo Fisher Scientific). Aliquots of 30 μg of proteins, denatured in buffer (20% glycerol, 10% β-mercaptoethanol, 10% sodium dodecyl sulfate [SDS], 62.5 mM Tris) were analyzed from 12% SDS–polyacrylamide gel electrophoresis (PAGE) and transferred to PVDF Immobilon membranes (Bio-Rad). After 1-hr saturation in TBS (Tris-Buffered Saline)/0.2% Tween/2% milk at room temperature, the membranes were probed (overnight at 4°C) with rabbit polyclonal anti-tyrosine hydroxylase (Merck, AB152) diluted in TBS/0.2% Tween/2% milk. Then, membranes were rinsed threetimes in TBS/0.2% Tween for 10 min and incubated for 1 hr with goat secondary anti-rabbit IgG linked to peroxidase (dilution 1:5000; Bio-Rad) in TBS/0.2% Tween/2% milk. Membranes were rinsed again and exposed to Clarity Western ECL Substrate (Bio-Rad). The intensity of the spots was determined by densitometry with ChemiDoc Software (Bio-Rad) and analyzed using the Image LabTM software (Bio-Rad). Quantification of whole protein levels (using the stain-free protocol provided by Bio-Rad) was used for normalization. Stain-Free technology enabled fluorescent visualization of 1D SDS–PAGE gels and corresponding blots. The relative amount of total protein in each lane on the blot was calculated and used for quantitation normalization.

### Histology

BAT and WAT samples were fixed in 10 ml Zinc Formal Fixx (Thermo Fisher Scientific) during 24 hr at 4°C. Samples were dehydrated using the Leica TP1020 semi-enclosed processor and embedded in paraffin. 6 μm sections were processed for immunohistochemistry (IHC) and EdU detection. Tissue sections from different mice were assayed on the same slide to minimize staining variability.

For IHC, deparaffinized sections were incubated overnight at 4°C with rabbit poly-clonal antibodies directed against UCP1 (Abcam, ab10983), diluted 1:400 in phosphate-buffered saline (PBS)/2.5% goat serum. Sections were then incubated with a horseradish peroxidase-labeled anti-rabbit antibody (1:300, Promega, W401B) for 1 hr at room temperature. Peroxidase activity was visualized with diaminobenzidine staining (Sigma-Aldrich, D5905). Images were acquired using an AxioObserver Zeiss microscope at a ×16 magnification.

EdU detection was assessed as recommended by the manufacturer (Click-iT EdU Cell Proliferation Kit, Thermo Fisher), following deparaffinization of BAT sections. Images were acquired on a DM6000 Leica microscope. Both EdU-positive cells and DAPI (4’,6-diamidino-2-phenylindol)-marked nuclei were quantified on pictures of whole BAT sections to avoid any bias of field selection.

### Plasma T3/T4 quantification

Plasmatic free T3 and free T4 were quantified on a Cobas 6000 automat with the Cobas e601module (Roche, ECL analysers).

### RNA extraction and RT-qPCR

RNAs were extracted using Trizol (Invitrogen, Carlsbad, CA, USA). Total RNA was reverse transcribed to cDNA using MMLV reverse transcriptase (Promega, Wisconsin, USA). RT-qPCRs (quantitative reverse transcription polymerase chain reactions) were performed using SYBRGreen mix (Bio-Rad iQ supermix). The results were analyzed according to the ΔΔCT method (Bookout and Mangelsdorf, 2003). *Hprt* was used as the reference gene. Primers are listed in Table 2.

### RNAseq analysis

cDNA libraries were prepared using the total RNA SENSE kit (Lexogen, Vienna, Austria) and analyzed on a Nextseq 500 sequencer (Illumina) as previously described (Richard et al., 2020; Guyot et al., 2014). Raw data of single-end sequencing were aligned on the GRCm38 (mm10) reference genome using Bowtie (Galaxy Version 2.2.6.2) and converted to count tables using htseqcount (Galaxy Version 0.6.1galaxy3), respectively. Differential gene expression analysis was performed with DESeq2 (R package, Version 1.34.0) (Love et al., 2014) using the following thresholds: adjusted p value <0.05; average expression >10 reads per million, log_2_ fold-change >0.6 or <−0.6. The effects of mutations on cold exposure were assessed using the interaction model (Ge, 2021). Thresholds for the interaction model were the following: average expression >10 reads per million, adjusted p value <0.05.

Differentially expressed genes expression was visualized as clustered heatmaps using Pheatmap R package (RRID:SCR_016418). Normalized counts from DESeq2 were used as inputs and the correlation method was used to cluster the genes. Data were scaled independently for each gene, with the same color code for all genes (red: above mean, white: mean, blue: below mean).

Gene ontology analyses were made using the Gene Ontology Resource (http://geneontology.org).

### ChIPseq and analysis

Freshly dissected small pieces of BAT were incubated during 25 min at room temperature under agitation with a crosslink solution (250 mM disuccinimidyl glutarate, 50 mM 4-(2-hydroxyethyl)-1-piperazineethanesulfonic acid, 100 mM NaCl, 1 mM EDTA, 0.5 mM ethylene glycol tetraacetic acid). Then, 1% formaldehyde was added for 20 min followed by 50 mM glycine addition. The tissue was rinsed several times with cold PBS. Chromatin immunoprecipitation was then performed as previously described (Chatonnet et al., 2013). Sequencing libraries were prepared from the immunoprecipitated fraction and the input fraction as a control, using the Accel-NGS 2S Plus DNA library kits with single indexing (Swift Biosciences). They were analyzed on a Nextseq 500 sequencer (Illumina). Raw data of paired-end sequencing were aligned on the GRCm38 (mm10) reference genome using Bowtie (Galaxy Version 2.2.6.2). MACS2 (Galaxy Version 2.1.1.20160309.0) was used for peak calling and peaks with a score inferior to 60 were filtered out. De novo motif search was performed using SeqPos motif tool (version 1.0.0). Genes within 30 kb of peaks were called out using GREAT (http://great.stanford.edu/public/html/). We chose a distance of 30 kb upstream or downstream of the TSS to attribute a TRBS to a gene. Although arbitrary, this distance was found to maximize the ratio of T3-responsive genes among the included genes, without excluding genes which have been well characterized as TRα1 target genes in other neural systems, such as *Klf9* or *Hr* (Gil-Ibañez et al., 2013). The distribution of distances of TRBSs around TSSs, as well as the distribution of TRBSs in the genome, were assessed using PAVIS (https://manticore.niehs.nih.gov/pavis2/).

### Statistical analysis

The data shown represent the average values for animals with the same genotype that were given the same treatment. The number of animals used in each experiment (*n*) is indicated in figure legends. Except when anything else is mentioned, the error bars represent the standard deviation. For comparing two means, the statistical relevance was determined using an unpaired Student’s *t*-test. For determining the effects of the mutations on food consumption, body temperature, body weight overtime, we used a two-way analysis of variance (ANOVA) with time as one factor, and the other parameter as the second factor. For comparing the different levels of one factor to a control group, the statistical relevance was determined using the one-way ANOVA method. When this test showed significant differences (p value <0.05), a post hoc Tukey test was used for multiple comparisons. To assess the effects of the mutations or the denervation on a given treatment (2 factors with 2 levels each), the statistical relevance was determined using a two-way ANOVA. Only when the interaction term was significant (interaction p value <0.05), a post hoc Tukey test was used to compare the effects of the indicated combinations. For any of these tests, statistical relevance is shown in the graphs as follows: *p value <0.05, **p value <0.01, ***p value <0.001.

## Data Availability

The raw sequencing data and aligned read counts generated as part of this study have been deposited to the NCBI Sequence Read Archive. Accession number: GSE201136; https://www.ncbi.nlm.nih.gov/geo/query/acc.cgi?acc=GSE201136. The following dataset was generated: ZekriY GuyotR FlamantF 2022Target genes of thyroid hormone (TH) in brown adipose tissue (BAT) and their role during metabolic stressorsNCBI Gene Expression OmnibusGSE201136 The following previously published dataset was used: ChangJS 2018A map of the PGC-1α- and NT-PGC-1α-regulated transcriptional network in brown adipose tissueNCBI Gene Expression OmnibusGSE11005310.1038/s41598-018-26244-4PMC595987029777200

## Additional files

MDAR checklist

## References

[bib1] Adams AC, Astapova I, Fisher FM, Badman MK, Kurgansky KE, Flier JS, Hollenberg AN, Maratos-Flier E (2010). Thyroid hormone regulates hepatic expression of fibroblast growth factor 21 in a pparalpha-dependent manner. The Journal of Biological Chemistry.

[bib2] Biagi CAO, Cury SS, Alves CP, Rabhi N, Silva WA, Farmer SR, Carvalho RF, Batista ML (2021). Multidimensional single-nuclei RNA-seq reconstruction of adipose tissue reveals adipocyte plasticity underlying thermogenic response. Cells.

[bib3] Bianco AC, Silva JE (1987). Intracellular conversion of thyroxine to triiodothyronine is required for the optimal thermogenic function of brown adipose tissue. The Journal of Clinical Investigation.

[bib4] Bianco AC, Sheng XY, Silva JE (1988). Triiodothyronine amplifies norepinephrine stimulation of uncoupling protein gene transcription by a mechanism not requiring protein synthesis. The Journal of Biological Chemistry.

[bib5] Bianco AC, Silva JE (1988). Cold exposure rapidly induces virtual saturation of brown adipose tissue nuclear T3 receptors. The American Journal of Physiology.

[bib6] Biondi B, Fazio S, Carella C, Amato G, Cittadini A, Lupoli G, Saccà L, Bellastella A, Lombardi G (1993). Cardiac effects of long term thyrotropin-suppressive therapy with levothyroxine. The Journal of Clinical Endocrinology and Metabolism.

[bib7] Bookout AL, Mangelsdorf DJ (2003). Quantitative real-time PCR protocol for analysis of nuclear receptor signaling pathways. Nuclear Receptor Signaling.

[bib8] Bredella MA, Fazeli PK, Freedman LM, Calder G, Lee H, Rosen CJ, Klibanski A (2012). Young women with cold-activated brown adipose tissue have higher bone mineral density and lower pref-1 than women without brown adipose tissue: a study in women with anorexia nervosa, women recovered from anorexia nervosa, and normal-weight women. The Journal of Clinical Endocrinology and Metabolism.

[bib9] Cannon B, Nedergaard J (2004). Brown adipose tissue: function and physiological significance. Physiological Reviews.

[bib10] Cao W, Daniel KW, Robidoux J, Puigserver P, Medvedev AV, Bai X, Floering LM, Spiegelman BM, Collins S (2004). P38 mitogen-activated protein kinase is the central regulator of cyclic AMP-dependent transcription of the brown fat uncoupling protein 1 gene. Molecular and Cellular Biology.

[bib11] Castillo M, Hall JA, Correa-Medina M, Ueta C, Kang HW, Cohen DE, Bianco AC (2011). Disruption of thyroid hormone activation in type 2 deiodinase knockout mice causes obesity with glucose intolerance and liver steatosis only at thermoneutrality. Diabetes.

[bib12] Challa TD, Dapito DH, Kulenkampff E, Kiehlmann E, Moser C, Straub L, Sun W, Wolfrum C (2020). A genetic model to study the contribution of brown and brite adipocytes to metabolism. Cell Reports.

[bib13] Chang JS, Ghosh S, Newman S, Salbaum JM (2018). A map of the PGC-1α- and NT-PGC-1α-regulated transcriptional network in brown adipose tissue. Scientific Reports.

[bib14] Chatonnet F, Guyot R, Benoît G, Flamant F (2013). Genome-wide analysis of thyroid hormone receptors shared and specific functions in neural cells. PNAS.

[bib15] Christoffolete MA, Linardi CCG, de Jesus L, Ebina KN, Carvalho SD, Ribeiro MO, Rabelo R, Curcio C, Martins L, Kimura ET, Bianco AC (2004). Mice with targeted disruption of the DIO2 gene have cold-induced overexpression of the uncoupling protein 1 gene but fail to increase brown adipose tissue lipogenesis and adaptive thermogenesis. Diabetes.

[bib16] de Jesus LA, Carvalho SD, Ribeiro MO, Schneider M, Kim SW, Harney JW, Larsen PR, Bianco AC (2001). The type 2 iodothyronine deiodinase is essential for adaptive thermogenesis in brown adipose tissue. The Journal of Clinical Investigation.

[bib17] De Leo S, Lee SY, Braverman Leh (2016). Hyperthyroidism. Lancet.

[bib18] Engelhard A, Christiano AM (2004). The hairless promoter is differentially regulated by thyroid hormone in keratinocytes and neuroblastoma cells. Experimental Dermatology.

[bib19] Fenzl A, Kiefer FW (2014). Brown adipose tissue and thermogenesis: horm mol biol clin investig. Horm Mol Biol Clin Investig.

[bib20] Finan B, Clemmensen C, Zhu Z, Stemmer K, Gauthier K, Müller L, De Angelis M, Moreth K, Neff F, Perez-Tilve D, Fischer K, Lutter D, Sánchez-Garrido MA, Liu P, Tuckermann J, Malehmir M, Healy ME, Weber A, Heikenwalder M, Jastroch M, Kleinert M, Jall S, Brandt S, Flamant F, Schramm KW, Biebermann H, Döring Y, Weber C, Habegger KM, Keuper M, Gelfanov V, Liu F, Köhrle J, Rozman J, Fuchs H, Gailus-Durner V, Hrabě de Angelis M, Hofmann SM, Yang B, Tschöp MH, DiMarchi R, Müller TD (2016). Chemical hybridization of glucagon and thyroid hormone optimizes therapeutic impact for metabolic disease. Cell.

[bib21] Flamant F (2016). Futures challenges in thyroid hormone signaling research. Frontiers in Endocrinology.

[bib22] Fonseca TL, Werneck-De-Castro JP, Castillo M, Bocco B, Fernandes GW, McAninch EA, Ignacio DL, Moises CCS, Ferreira AR, Ferreira A, Gereben B, Bianco AC (2014). Tissue-specific inactivation of type 2 deiodinase reveals multilevel control of fatty acid oxidation by thyroid hormone in the mouse. Diabetes.

[bib23] Fukano K, Okamatsu-Ogura Y, Tsubota A, Nio-Kobayashi J, Kimura K (2016). Cold exposure induces proliferation of mature brown adipocyte in a ß3-adrenergic receptor-mediated pathway. PLOS ONE.

[bib24] Ge X (2021). IDEP web application for RNA-seq data analysis. Methods in Molecular Biology.

[bib25] Gil-Ibañez P, Morte B, Bernal J (2013). Role of thyroid hormone receptor subtypes α and β on gene expression in the cerebral cortex and striatum of postnatal mice. Endocrinology.

[bib26] Guyot R, Chatonnet F, Gillet B, Hughes S, Flamant F (2014). Toxicogenomic analysis of the ability of brominated flame retardants TBBPA and BDE-209 to disrupt thyroid hormone signaling in neural cells. Toxicology.

[bib27] Hall JA, Ribich S, Christoffolete MA, Simovic G, Correa-Medina M, Patti ME, Bianco AC (2010). Absence of thyroid hormone activation during development underlies a permanent defect in adaptive thermogenesis. Endocrinology.

[bib28] Hankir MK, Klingenspor M (2018). Brown adipocyte glucose metabolism: a heated subject. EMBO Reports.

[bib29] Hirose K, Payumo AY, Cutie S, Hoang A, Zhang H, Guyot R, Lunn D, Bigley RB, Yu H, Wang J, Smith M, Gillett E, Muroy SE, Schmid T, Wilson E, Field KA, Reeder DM, Maden M, Yartsev MM, Wolfgang MJ, Grützner F, Scanlan TS, Szweda LI, Buffenstein R, Hu G, Flamant F, Olgin JE, Huang GN (2019). Evidence for hormonal control of heart regenerative capacity during endothermy acquisition. Science.

[bib30] Jeong JH, Chang JS, Jo YH (2018). Intracellular glycolysis in brown adipose tissue is essential for optogenetically induced nonshivering thermogenesis in mice. Scientific Reports.

[bib31] Lahesmaa M, Orava J, Schalin-Jäntti C, Soinio M, Hannukainen JC, Noponen T, Kirjavainen A, Iida H, Kudomi N, Enerbäck S, Virtanen KA, Nuutila P (2014). Hyperthyroidism increases brown fat metabolism in humans. The Journal of Clinical Endocrinology and Metabolism.

[bib32] Liu S, Shen S, Yan Y, Sun C, Lu Z, Feng H, Ma Y, Tang Z, Yu J, Wu Y, Gereben B, Mohácsik P, Fekete C, Feng X, Yuan F, Guo F, Hu C, Shao M, Gao X, Zhao L, Li Y, Jiang J, Ying H (2022). Triiodothyronine (T3) promotes brown fat hyperplasia via thyroid hormone receptor α mediated adipocyte progenitor cell proliferation. Nature Communications.

[bib33] López M, Varela L, Vázquez MJ, Rodríguez-Cuenca S, González CR, Velagapudi VR, Morgan DA, Schoenmakers E, Agassandian K, Lage R (2010). A. Hypothalamic AMPK and Fatty Acid Metabolism Mediate Thyroid Regulation of Energy Balance. Nat Med.

[bib34] Love MI, Huber W, Anders S (2014). Moderated estimation of fold change and dispersion for RNA-seq data with deseq2. Genome Biology.

[bib35] Madisen L, Zwingman TA, Sunkin SM, Oh SW, Zariwala HA, Gu H, Ng LL, Palmiter RD, Hawrylycz MJ, Jones AR, Lein ES, Zeng H (2010). A robust and high-throughput CRE reporting and characterization system for the whole mouse brain. Nature Neuroscience.

[bib36] Martinez de Mena R, Scanlan TS, Obregon MJ (2010). The T3 receptor beta1 isoform regulates UCP1 and D2 deiodinase in rat brown adipocytes. Endocrinology.

[bib37] Martínez-Sánchez N, Moreno-Navarrete JM, Contreras C, Rial-Pensado E, Fernø J, Nogueiras R, Diéguez C, Fernández-Real JM, López M (2017). Thyroid hormones induce browning of white fat. The Journal of Endocrinology.

[bib38] Maushart CI, Loeliger R, Gashi G, Christ-Crain M, Betz MJ (2019). Resolution of hypothyroidism restores cold-induced thermogenesis in humans. Thyroid.

[bib39] Medina-Gomez G, Calvo RM, Obregon MJ (2008). Thermogenic effect of triiodothyroacetic acid at low doses in rat adipose tissue without adverse side effects in the thyroid axis. American Journal of Physiology. Endocrinology and Metabolism.

[bib40] Minakhina S, Bansal S, Zhang A, Brotherton M, Janodia R, De Oliveira V, Tadepalli S, Wondisford FE (2020). A direct comparison of thyroid hormone receptor protein levels in mice provides unexpected insights into thyroid hormone action. Thyroid.

[bib41] Nicolaisen TS, Klein AB, Dmytriyeva O, Lund J, Ingerslev LR, Fritzen AM, Carl CS, Lundsgaard AM, Frost M, Ma T, Schjerling P, Gerhart-Hines Z, Flamant F, Gauthier K, Larsen S, Richter EA, Kiens B, Clemmensen C (2020). Thyroid hormone receptor α in skeletal muscle is essential for T3-mediated increase in energy expenditure. FASEB Journal.

[bib42] Nitta A, Furukawa Y, Hayashi K, Hiramatsu M, Kameyama T, Hasegawa T, Nabeshima T (1992). Denervation of dopaminergic neurons with 6-hydroxydopamine increases nerve growth factor content in rat brain. Neuroscience Letters.

[bib43] Oppenheimer JH, Schwartz HL, Lane JT, Thompson MP (1991). Functional relationship of thyroid hormone-induced lipogenesis, lipolysis, and thermogenesis in the rat. The Journal of Clinical Investigation.

[bib44] Pascual A, Aranda A (2013). Thyroid hormone receptors, cell growth and differentiation. Biochimica et Biophysica Acta.

[bib45] Quignodon L, Vincent S, Winter H, Samarut J, Flamant F (2007). A point mutation in the activation function 2 domain of thyroid hormone receptor alpha1 expressed after cre-mediated recombination partially recapitulates hypothyroidism. Molecular Endocrinology.

[bib46] Reitman ML (2018). Of mice and men-environmental temperature, body temperature, and treatment of obesity. FEBS Letters.

[bib47] Richard S, Guyot R, Rey-Millet M, Prieux M, Markossian S, Aubert D, Flamant F (2020). A pivotal genetic program controlled by thyroid hormone during the maturation of gabaergic neurons. IScience.

[bib48] Ritter MJ, Amano I, Hollenberg AN (2020). Thyroid hormone signaling and the liver. Hepatology.

[bib49] Rosenwald M, Perdikari A, Rülicke T, Wolfrum C (2013). Bi-directional interconversion of brite and white adipocytes. Nature Cell Biology.

[bib50] Sáenz de Urturi D, Buqué X, Porteiro B, Folgueira C, Mora A, Delgado TC, Prieto-Fernández E, Olaizola P, Gómez-Santos B, Apodaka-Biguri M, González-Romero F, Nieva-Zuluaga A, Ruiz de Gauna M, Goikoetxea-Usandizaga N, García-Rodríguez JL, Gutierrez de Juan V, Aurrekoetxea I, Montalvo-Romeral V, Novoa EM, Martín-Guerrero I, Varela-Rey M, Bhanot S, Lee R, Banales JM, Syn WK, Sabio G, Martínez-Chantar ML, Nogueiras R, Aspichueta P (2022). Methionine adenosyltransferase 1A antisense oligonucleotides activate the liver-brown adipose tissue axis preventing obesity and associated hepatosteatosis. Nature Communications.

[bib51] Schneider MJ, Fiering SN, Pallud SE, Parlow AF, Galton VA (2001). Targeted disruption of the type 2 selenodeiodinase gene (DIO2) results in a phenotype of pituitary resistance to T4. Molecular Endocrinology.

[bib52] Silva JE, Larsen PR (1985). Potential of brown adipose tissue type II thyroxine 5’-deiodinase as a local and systemic source of triiodothyronine in rats. The Journal of Clinical Investigation.

[bib53] Silvestri E, Senese R, De Matteis R, Cioffi F, Moreno M, Lanni A, Gentile A, Busiello RA, Salzano AM, Scaloni A, de Lange P, Goglia F, Lombardi A (2020). Absence of uncoupling protein 3 at thermoneutrality influences brown adipose tissue mitochondrial functionality in mice. FASEB Journal.

[bib54] Škop V, Guo J, Liu N, Xiao C, Hall KD, Gavrilova O, Reitman ML (2020). Mouse thermoregulation: introducing the concept of the thermoneutral point. Cell Reports.

[bib55] Sun Y, Rahbani JF, Jedrychowski MP, Riley CL, Vidoni S, Bogoslavski D, Hu B, Dumesic PA, Zeng X, Wang AB, Knudsen NH, Kim CR, Marasciullo A, Millán JL, Chouchani ET, Kazak L, Spiegelman BM (2021). Mitochondrial TNAP controls thermogenesis by hydrolysis of phosphocreatine. Nature.

[bib56] Tabuchi C, Sul HS (2021). Signaling pathways regulating thermogenesis. Frontiers in Endocrinology.

[bib57] Uldry M, Yang W, St-Pierre J, Lin J, Seale P, Spiegelman BM (2006). Complementary action of the PGC-1 coactivators in mitochondrial biogenesis and brown fat differentiation. Cell Metabolism.

[bib58] Weir JB (1990). New methods for calculating metabolic rate with special reference to protein metabolism. 1949. J Physiol.

[bib59] Weiss RE, Murata Y, Cua K, Hayashi Y, Seo H, Refetoff S (1998). Thyroid hormone action on liver, heart, and energy expenditure in thyroid hormone receptor beta-deficient mice. Endocrinology.

[bib60] Whitfield ML, Sherlock G, Saldanha AJ, Murray JI, Ball CA, Alexander KE, Matese JC, Perou CM, Hurt MM, Brown PO, Botstein D (2002). Identification of genes periodically expressed in the human cell cycle and their expression in tumors. Molecular Biology of the Cell.

[bib61] Winter H, Rüttiger L, Müller M, Kuhn S, Brandt N, Zimmermann U, Hirt B, Bress A, Sausbier M, Conscience A, Flamant F, Tian Y, Zuo J, Pfister M, Ruth P, Löwenheim H, Samarut J, Engel J, Knipper M (2009). Deafness in trbeta mutants is caused by malformation of the tectorial membrane. The Journal of Neuroscience.

[bib62] Wolf M, Weigert A, Kreymann G (1996). Body composition and energy expenditure in thyroidectomized patients during short-term hypothyroidism and thyrotropin-suppressive thyroxine therapy. European Journal of Endocrinology.

[bib63] Wulf A, Harneit A, Kröger M, Kebenko M, Wetzel MG, Weitzel JM (2008). T3-mediated expression of PGC-1α via a far upstream located thyroid hormone response element. Molecular and Cellular Endocrinology.

[bib64] Yuan C, Nguyen P, Baxter JD, Webb P (2013). Distinct ligand-dependent and independent modes of thyroid hormone receptor (TR) /PGC-1α interaction. The Journal of Steroid Biochemistry and Molecular Biology.

[bib65] Zekri Y, Guyot R, Flamant F (2022). An atlas of thyroid hormone receptors’ target genes in mouse tissues. International Journal of Molecular Sciences.

