# Supplementary material for: Brown adipocytes local response to thyroid hormone is required for adaptive thermogenesis in adult male mice
Source: eLife. 2022 Nov 14;11:e81996. doi: 10.7554/eLife.81996 (PMC9683793; doi:10.7554/eLife.81996)
Supplement: Figure 2—source data 2. — Each lane represents a different sample, from sham mice (+) or denervated mice (−). [file elife-81996-fig2-data2.zip › Figure 2 - Source Data 1/Figure 2 - Source data 1.pptx]

## Slide 1
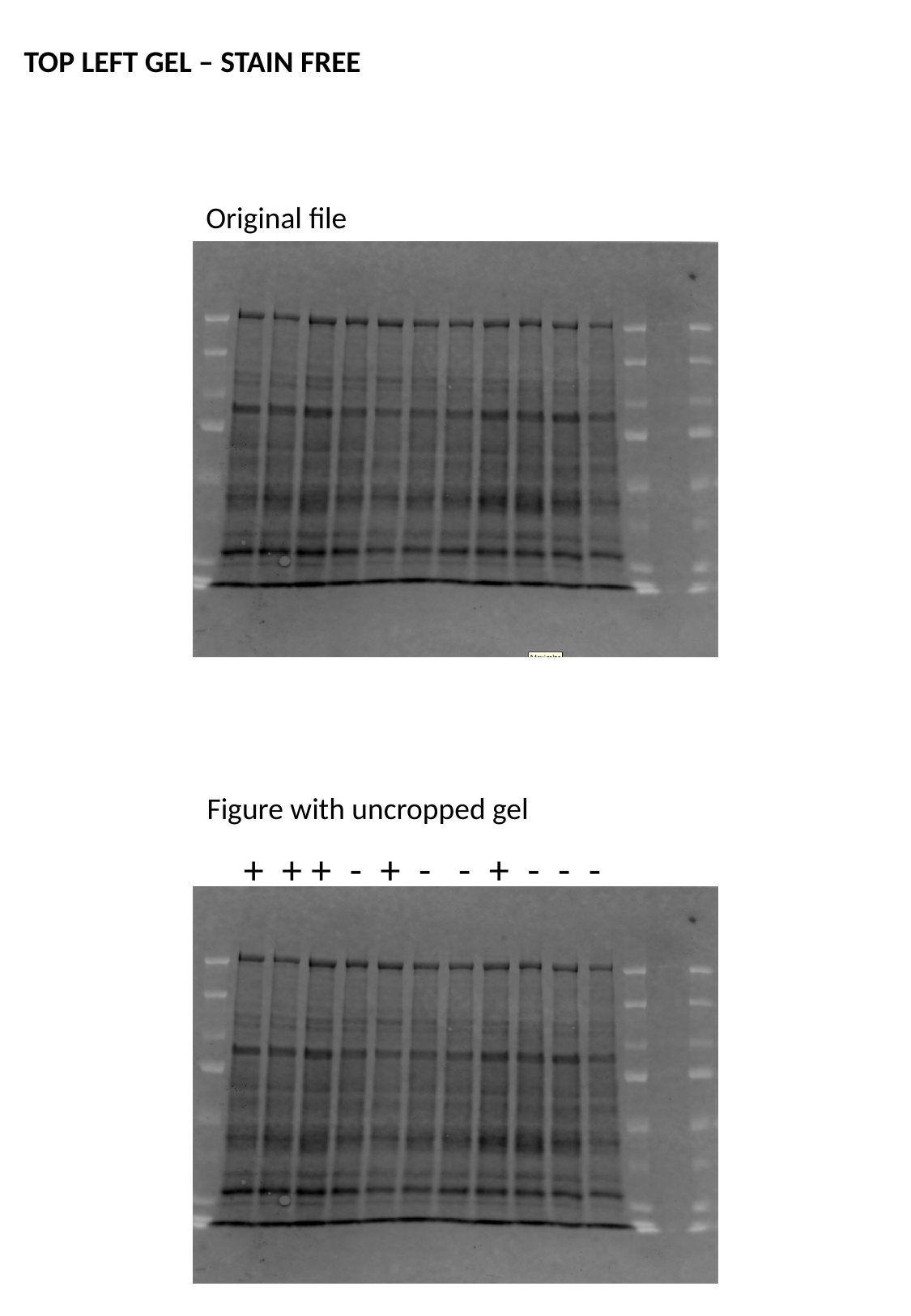

TOP LEFT GEL – STAIN FREE
Original file
Figure with uncropped gel
+ + + - + - - + - - -

## Slide 2
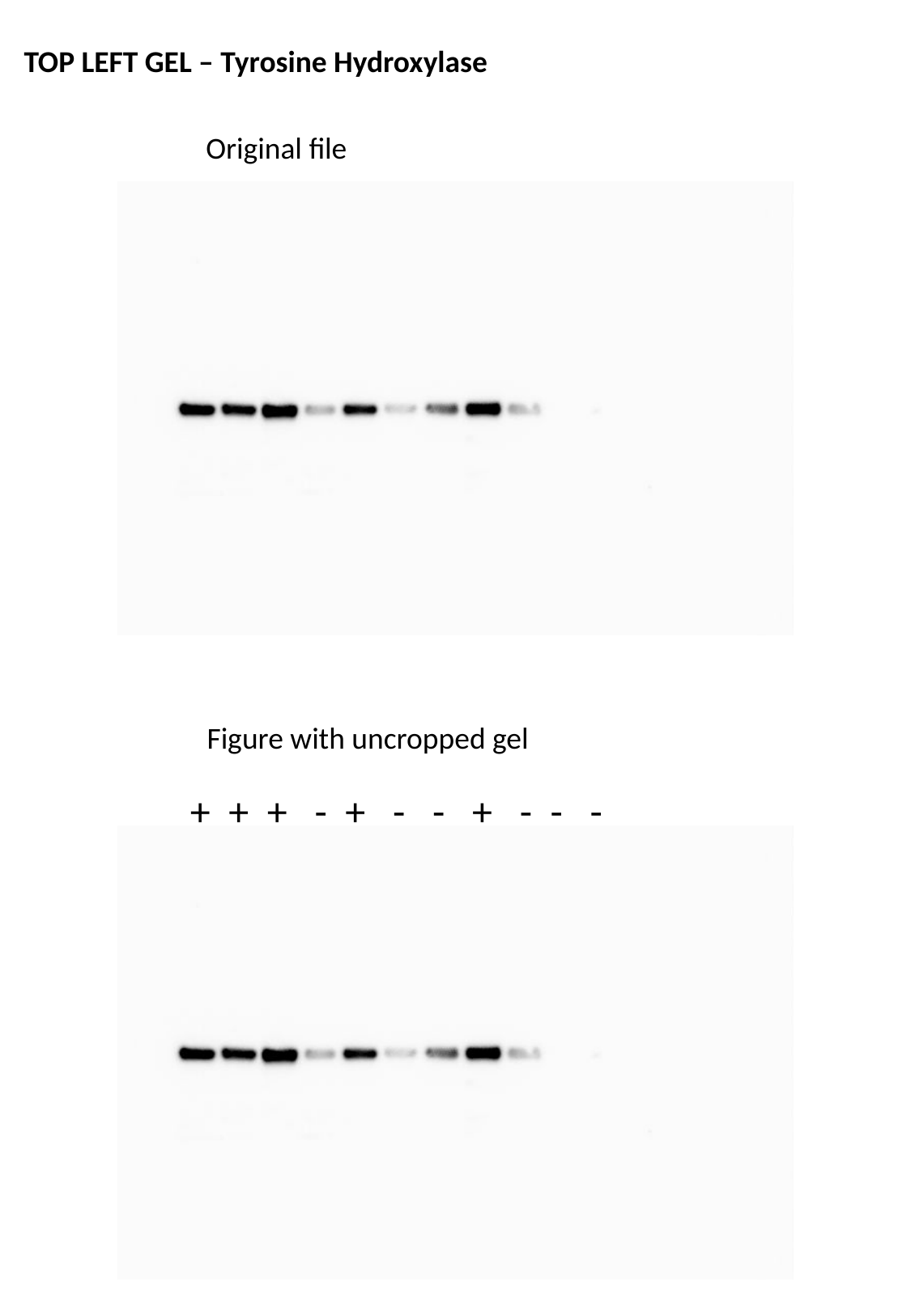

TOP LEFT GEL – Tyrosine Hydroxylase
Original file
Figure with uncropped gel
+ + + - + - - + - - -

## Slide 3
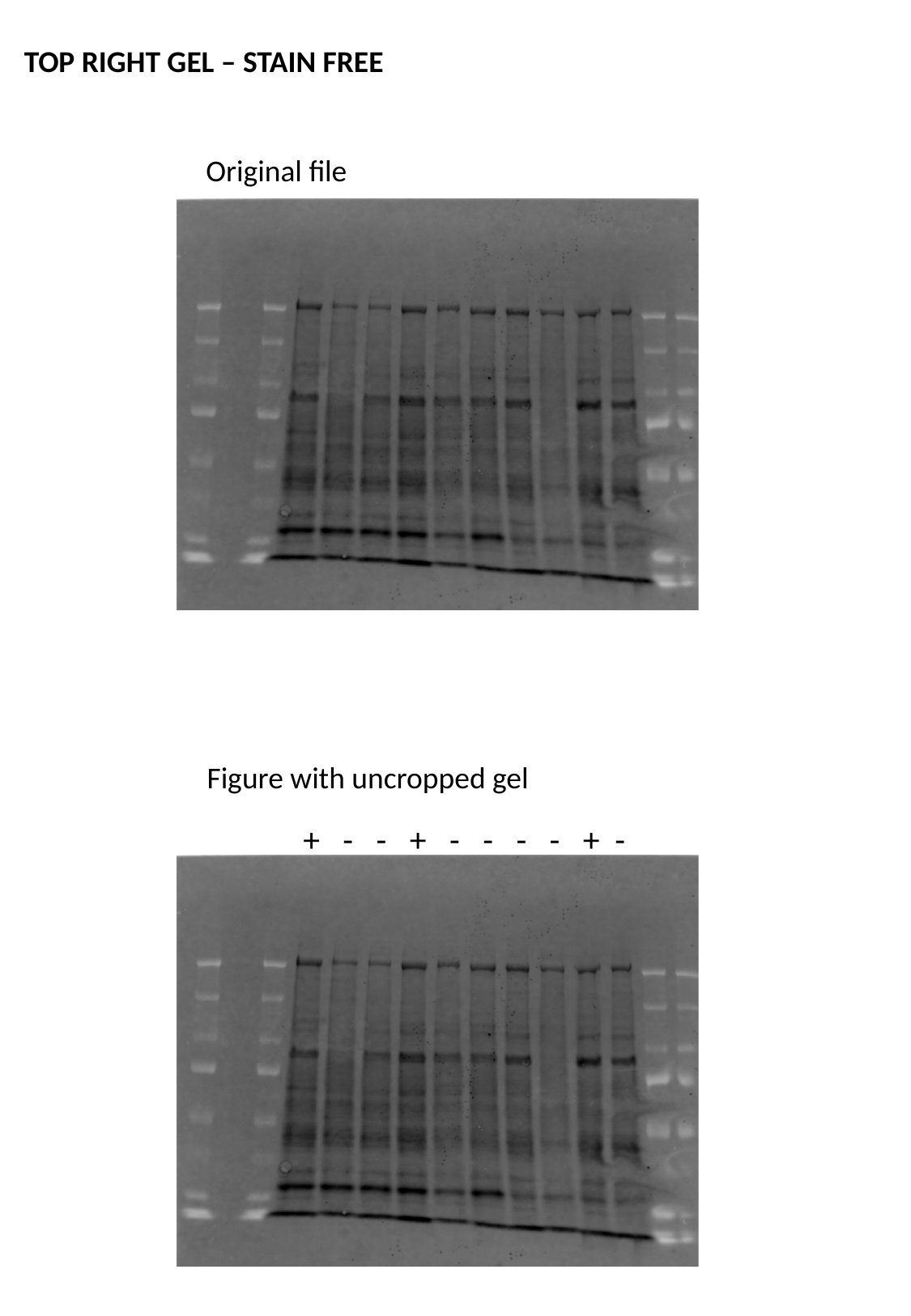

TOP RIGHT GEL – STAIN FREE
Original file
Figure with uncropped gel
+ - - + - - - - + -

## Slide 4
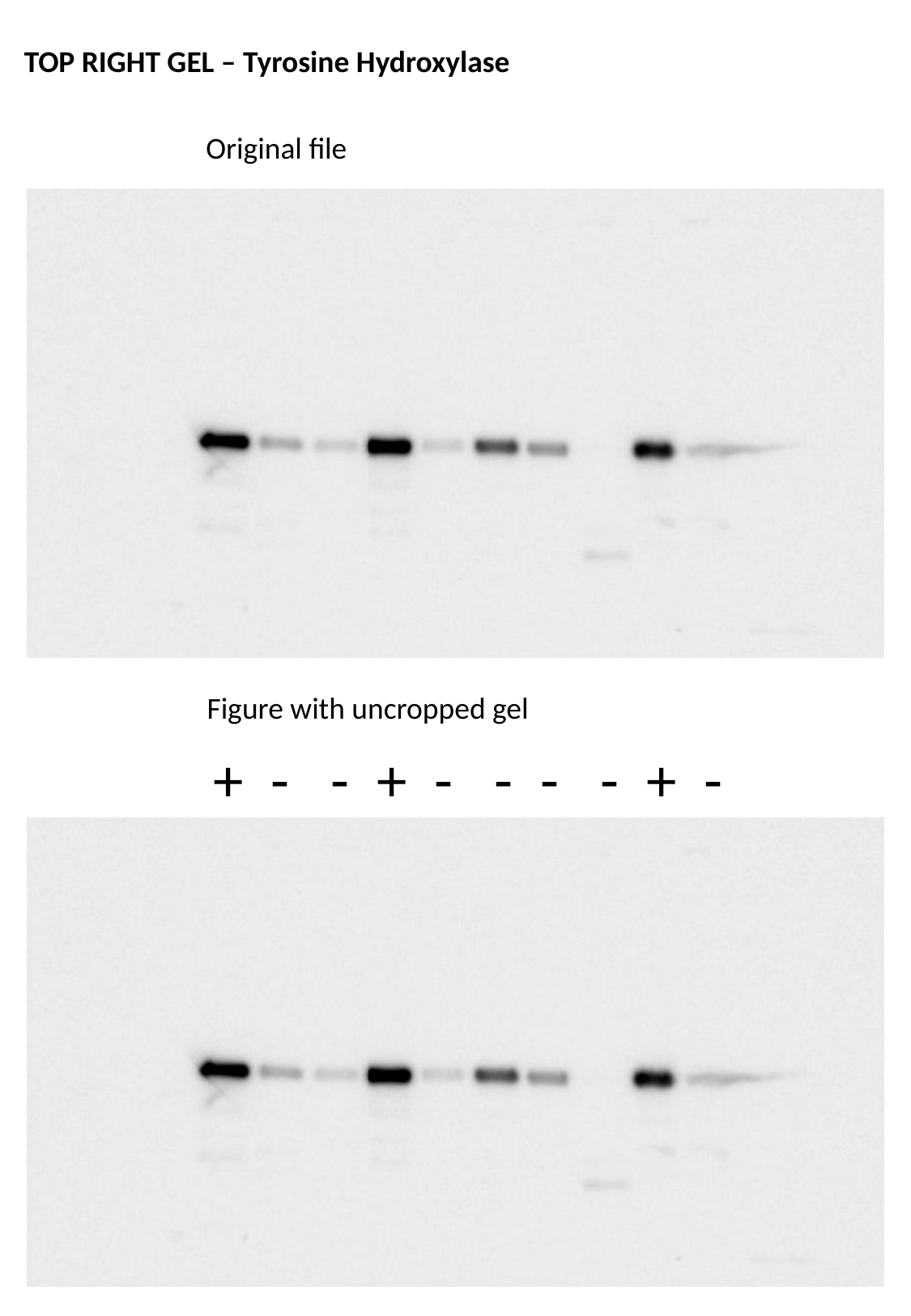

TOP RIGHT GEL – Tyrosine Hydroxylase
Original file
Figure with uncropped gel
+ - - + - - - - + -

## Slide 5
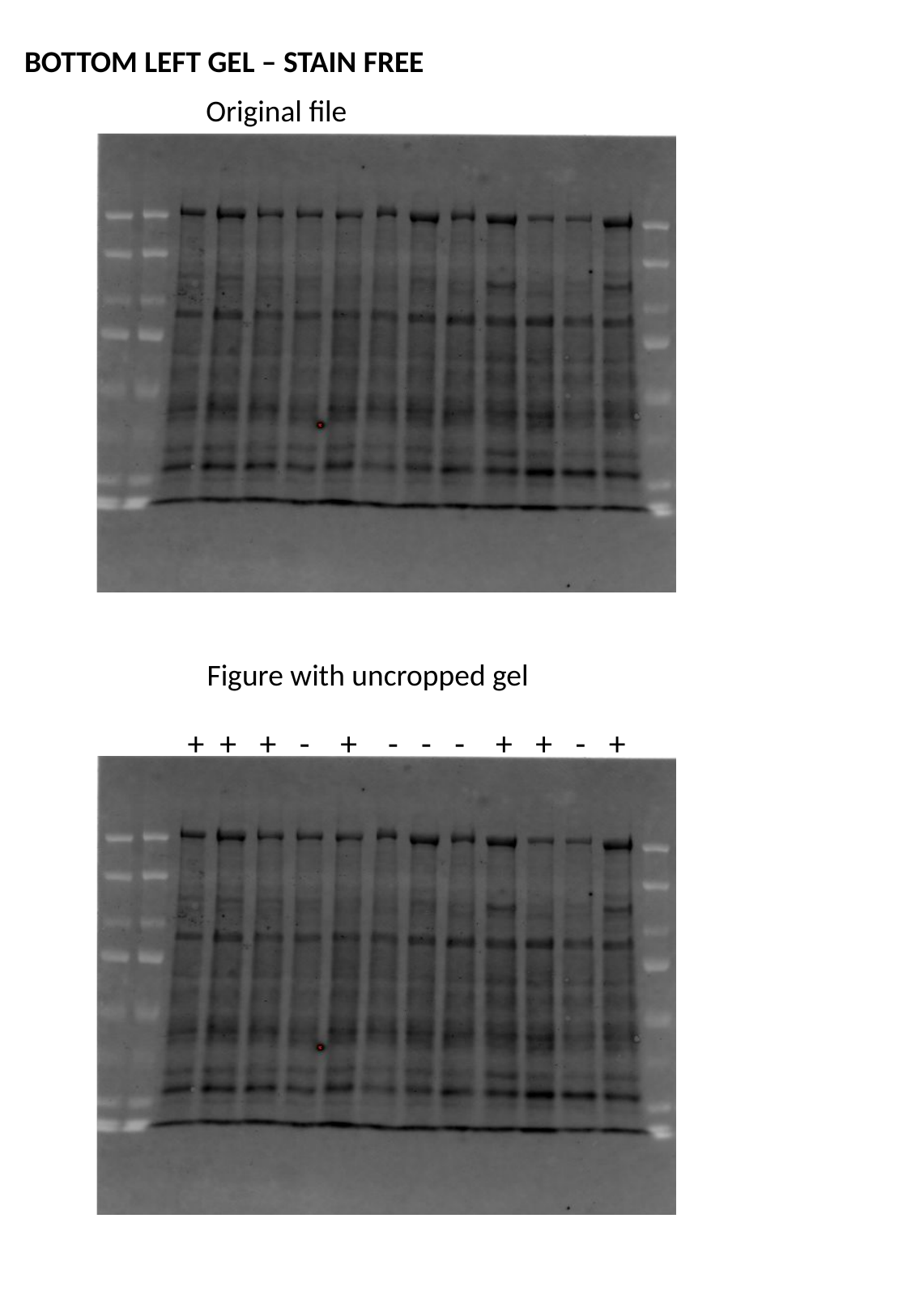

BOTTOM LEFT GEL – STAIN FREE
Original file
Figure with uncropped gel
+ + + - + - - - + + - +

## Slide 6
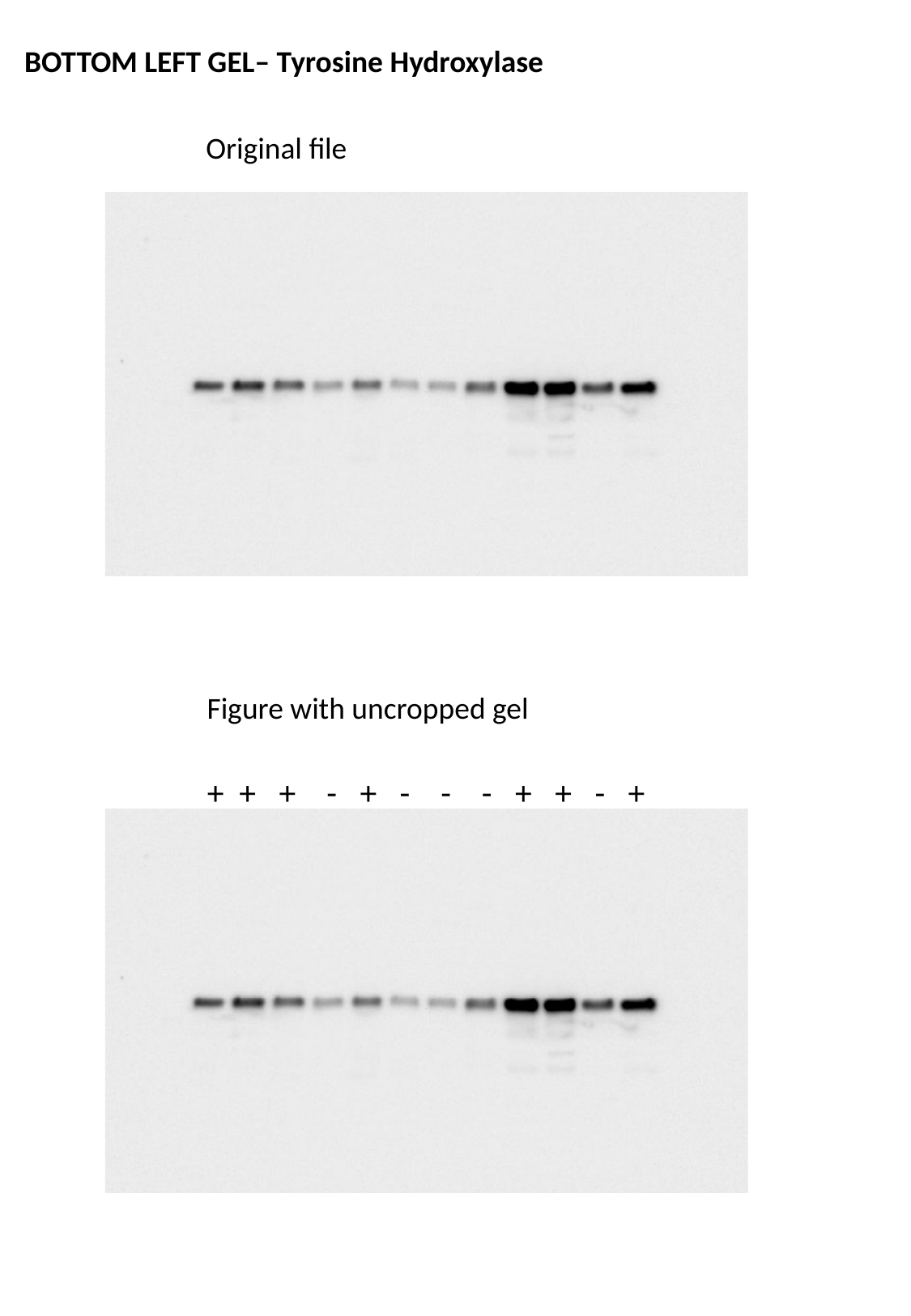

BOTTOM LEFT GEL– Tyrosine Hydroxylase
Original file
Figure with uncropped gel
+ + + - + - - - + + - +

## Slide 7
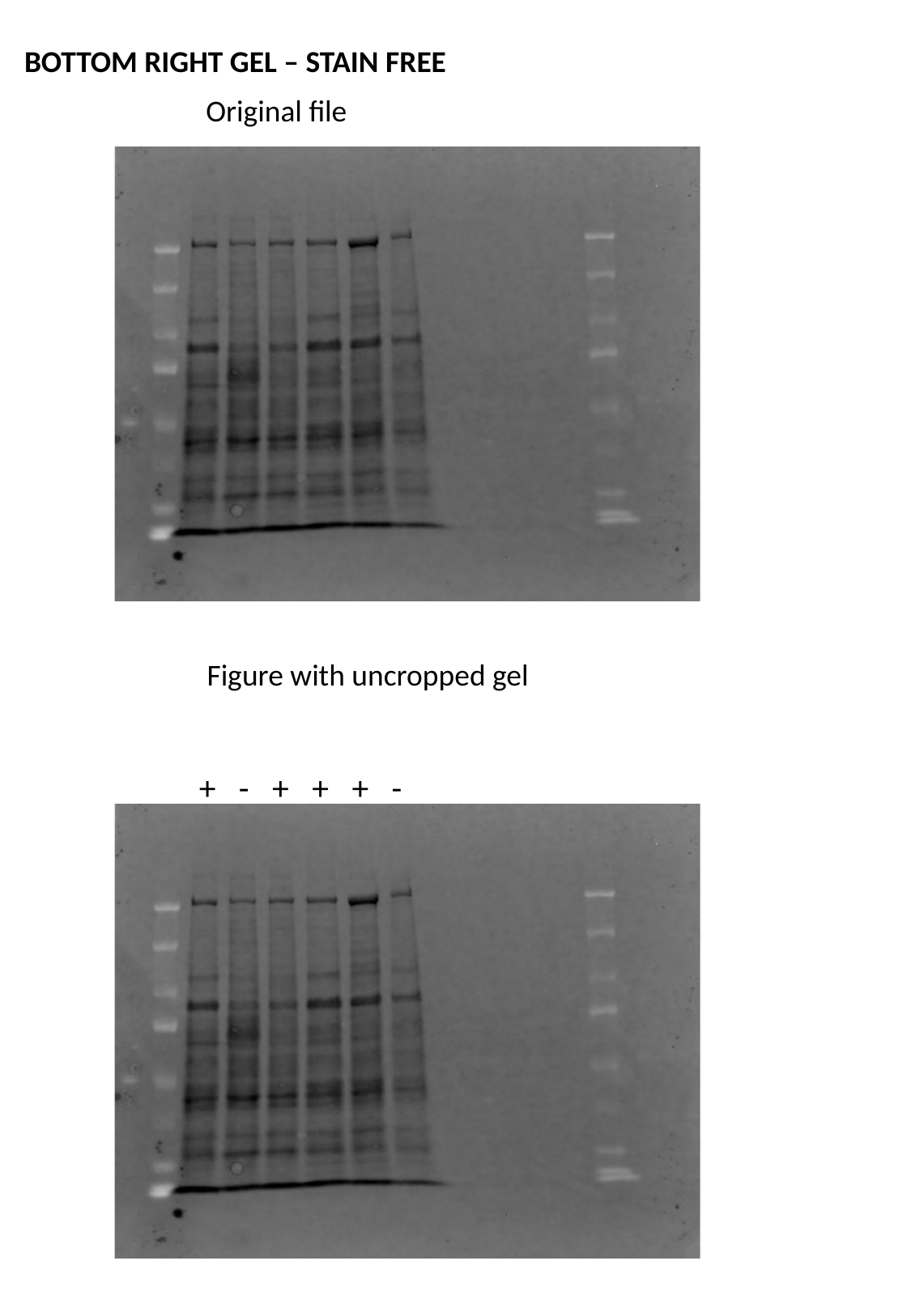

BOTTOM RIGHT GEL – STAIN FREE
Original file
Figure with uncropped gel
+ - + + + -

## Slide 8
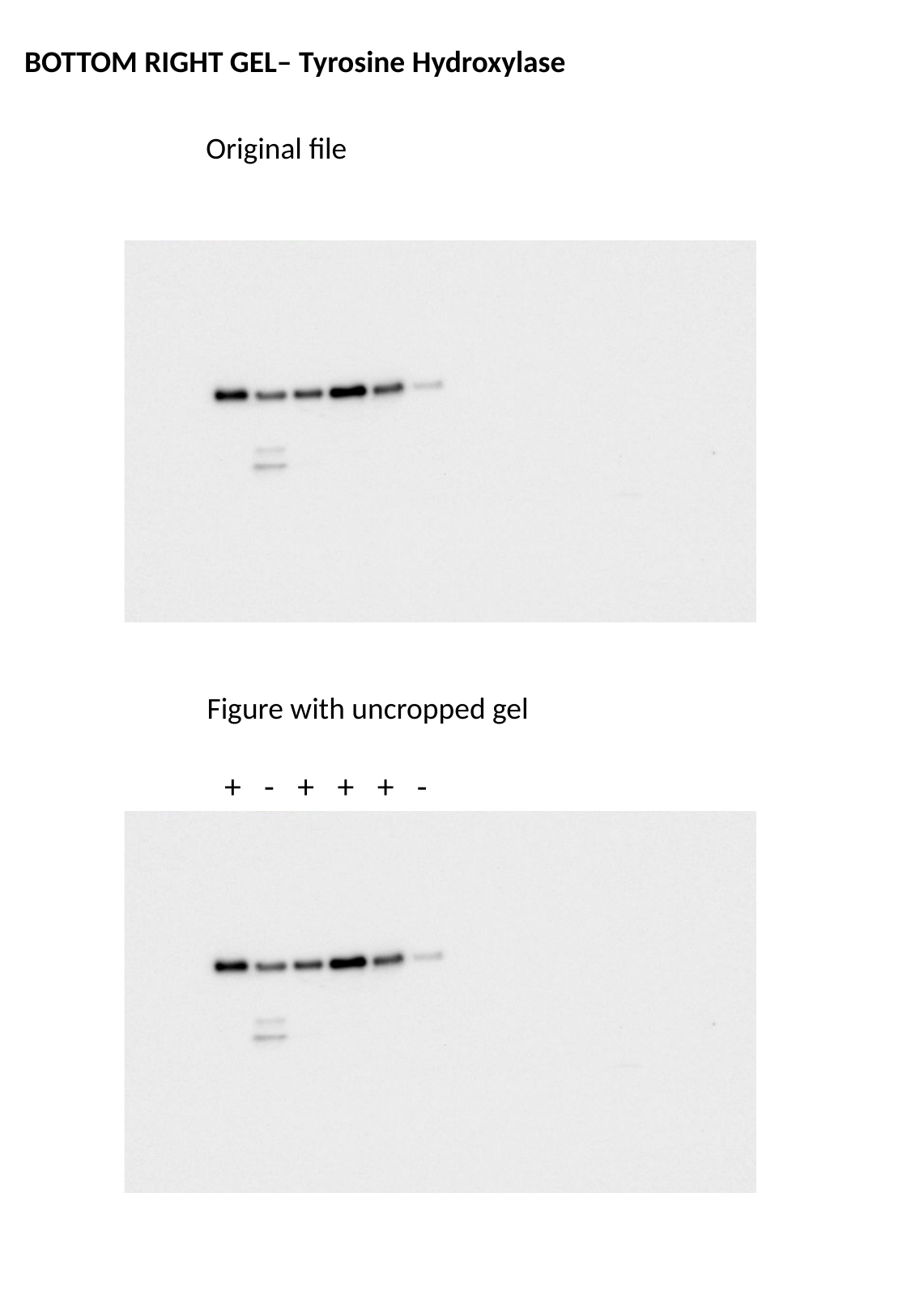

BOTTOM RIGHT GEL– Tyrosine Hydroxylase
Original file
Figure with uncropped gel
+ - + + + -
