# Supplementary figures and images for: Brown adipocytes local response to thyroid hormone is required for adaptive thermogenesis in adult male mice
Source: eLife. 2022 Nov 14;11:e81996. doi: 10.7554/eLife.81996 (PMC9683793; doi:10.7554/eLife.81996)

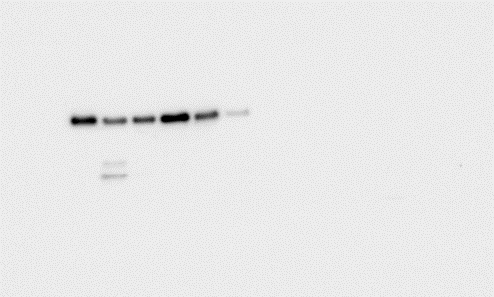

Supplement: Figure 2—source data 2. — Each lane represents a different sample, from sham mice (+) or denervated mice (−). [file elife-81996-fig2-data2.zip › Figure 2 - Source Data 1/Figure 2 - Source data 1 - BottomRight-TyrHydoxylase.png]

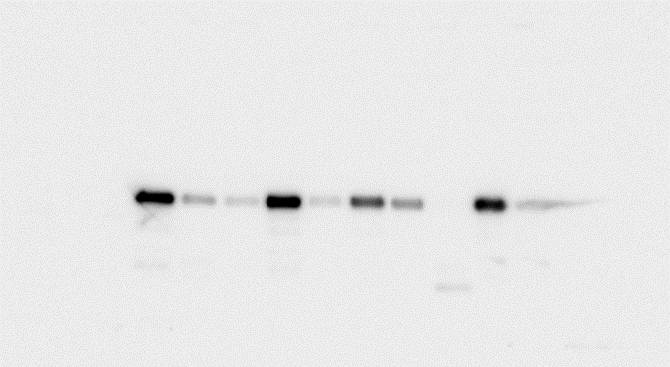

Supplement: Figure 2—source data 2. — Each lane represents a different sample, from sham mice (+) or denervated mice (−). [file elife-81996-fig2-data2.zip › Figure 2 - Source Data 1/Figure 2 - Source data 1 - TopRight-TyrHydoxylase.png]

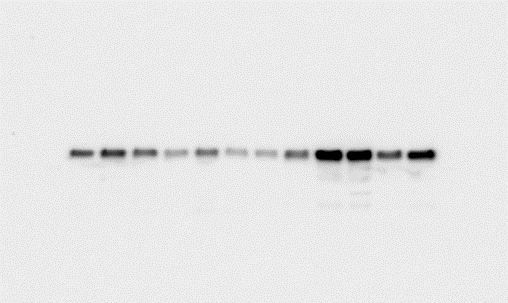

Supplement: Figure 2—source data 2. — Each lane represents a different sample, from sham mice (+) or denervated mice (−). [file elife-81996-fig2-data2.zip › Figure 2 - Source Data 1/Figure 2 - Source data 1 - BottomLeft-TyrHydoxylase.png]

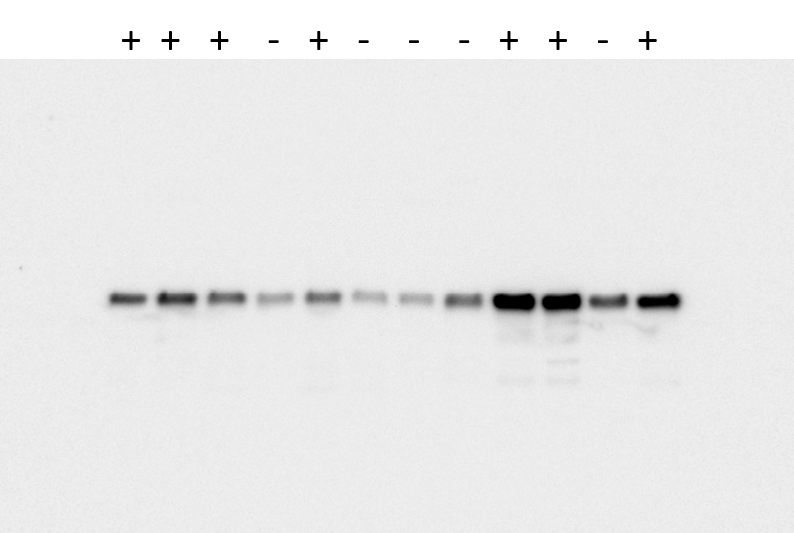

Supplement: Figure 2—source data 2. — Each lane represents a different sample, from sham mice (+) or denervated mice (−). [file elife-81996-fig2-data2.zip › Figure 2 - Source Data 1/Figure 2 - Source data 1 - BottomLeft-TyrHydoxylase_uncropped.png]

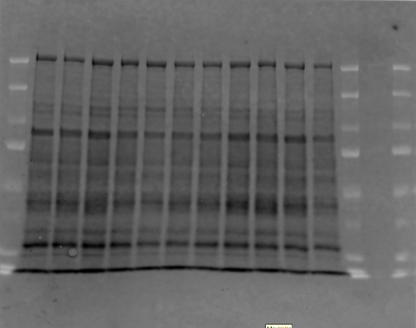

Supplement: Figure 2—source data 2. — Each lane represents a different sample, from sham mice (+) or denervated mice (−). [file elife-81996-fig2-data2.zip › Figure 2 - Source Data 1/Figure 2 - Source data 1 - TopLeft-StainFree.png]

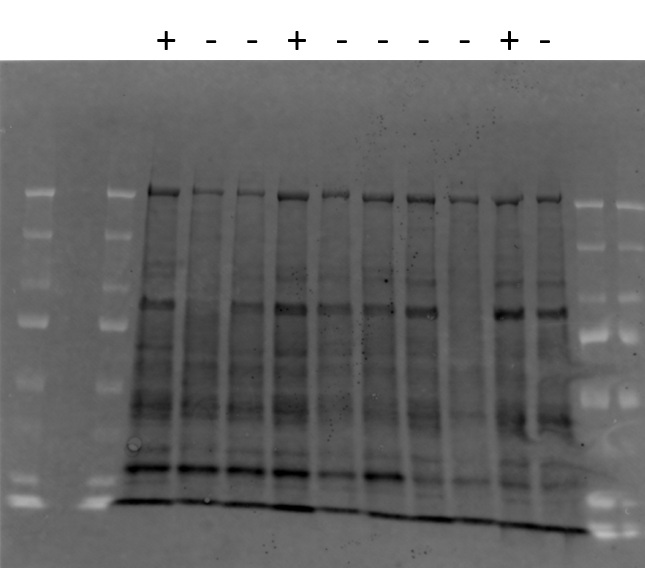

Supplement: Figure 2—source data 2. — Each lane represents a different sample, from sham mice (+) or denervated mice (−). [file elife-81996-fig2-data2.zip › Figure 2 - Source Data 1/Figure 2 - Source data 1 - TopRight-StainFree_uncropped.png]

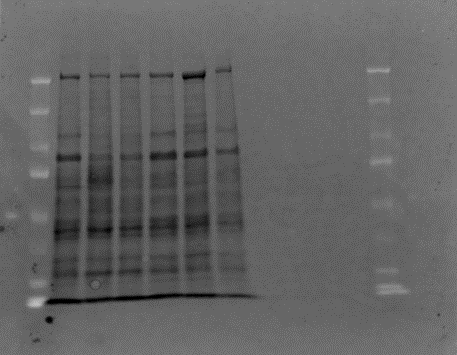

Supplement: Figure 2—source data 2. — Each lane represents a different sample, from sham mice (+) or denervated mice (−). [file elife-81996-fig2-data2.zip › Figure 2 - Source Data 1/Figure 2 - Source data 1 - BottomRight-StainFree.png]

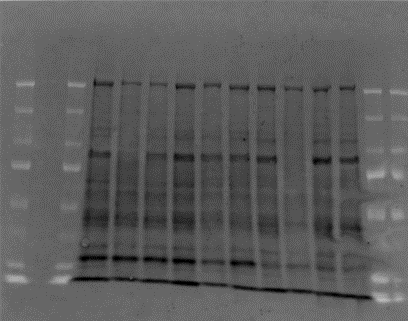

Supplement: Figure 2—source data 2. — Each lane represents a different sample, from sham mice (+) or denervated mice (−). [file elife-81996-fig2-data2.zip › Figure 2 - Source Data 1/Figure 2 - Source data 1 - TopRight-StainFree.png]

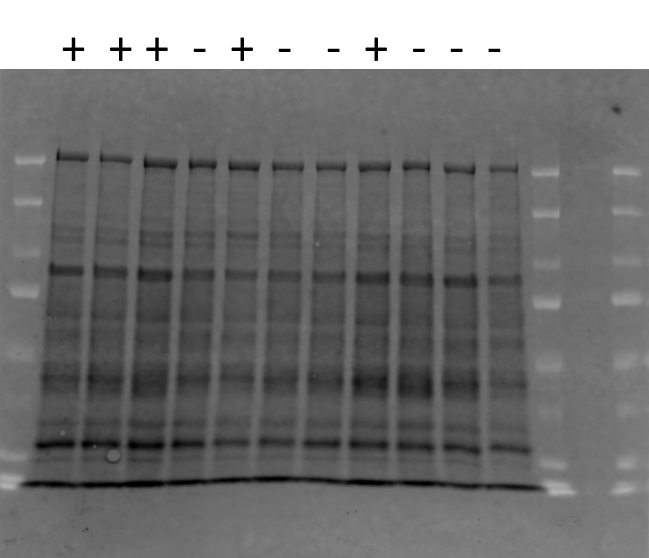

Supplement: Figure 2—source data 2. — Each lane represents a different sample, from sham mice (+) or denervated mice (−). [file elife-81996-fig2-data2.zip › Figure 2 - Source Data 1/Figure 2 - Source data 1 - TopLeft-StainFree_uncropped.png]

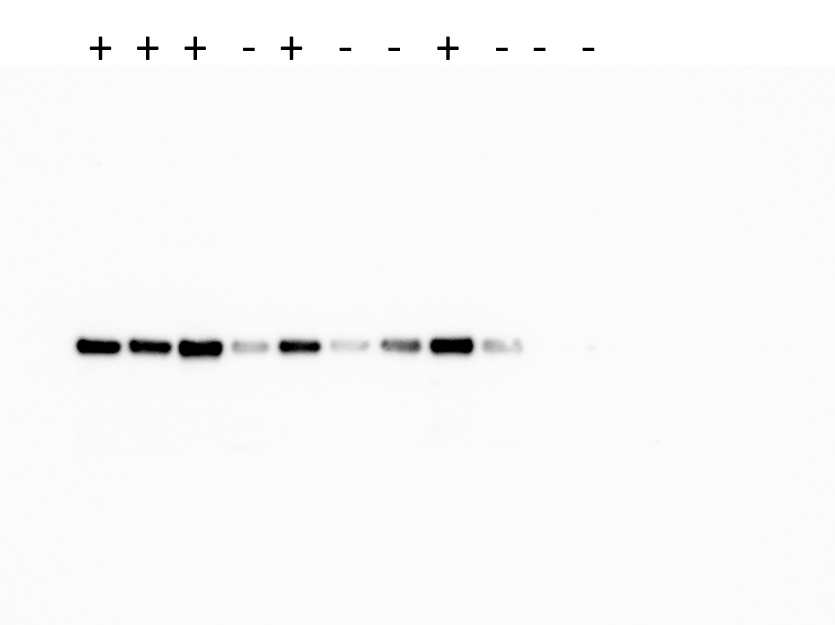

Supplement: Figure 2—source data 2. — Each lane represents a different sample, from sham mice (+) or denervated mice (−). [file elife-81996-fig2-data2.zip › Figure 2 - Source Data 1/Figure 2 - Source data 1 - TopLeft-TyrHydoxylase_uncropped.png]

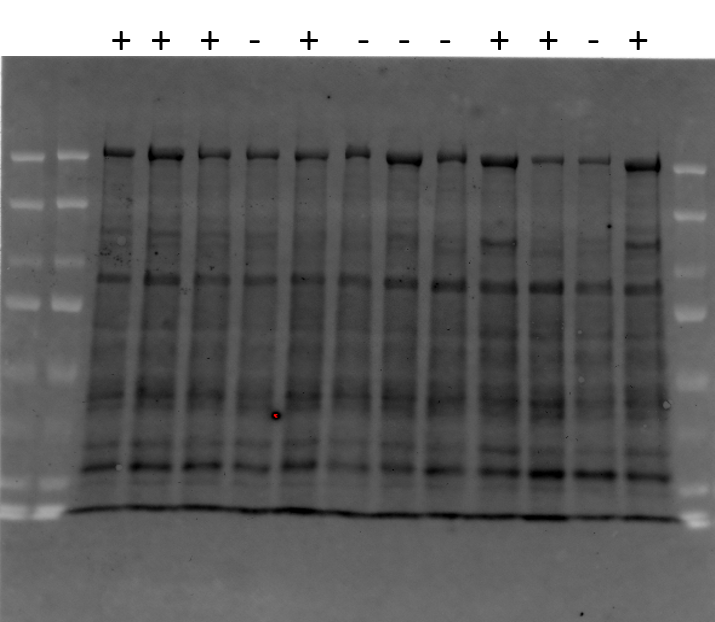

Supplement: Figure 2—source data 2. — Each lane represents a different sample, from sham mice (+) or denervated mice (−). [file elife-81996-fig2-data2.zip › Figure 2 - Source Data 1/Figure 2 - Source data 1 - BottomLeft-StainFree_uncropped.png]

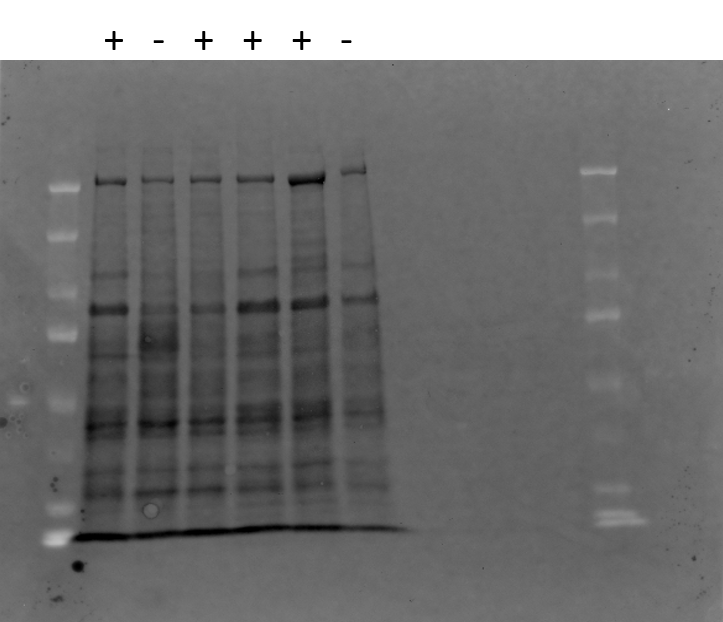

Supplement: Figure 2—source data 2. — Each lane represents a different sample, from sham mice (+) or denervated mice (−). [file elife-81996-fig2-data2.zip › Figure 2 - Source Data 1/Figure 2 - Source data 1 - BottomRight-StainFree_uncropped.png]

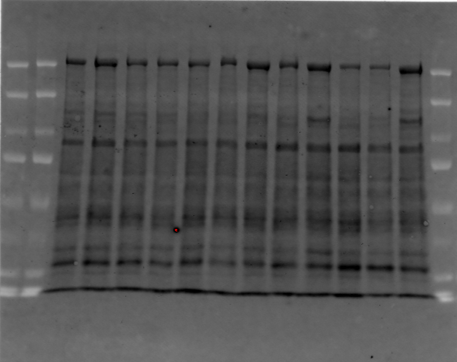

Supplement: Figure 2—source data 2. — Each lane represents a different sample, from sham mice (+) or denervated mice (−). [file elife-81996-fig2-data2.zip › Figure 2 - Source Data 1/Figure 2 - Source data 1 - BottomLeft-StainFree.png]

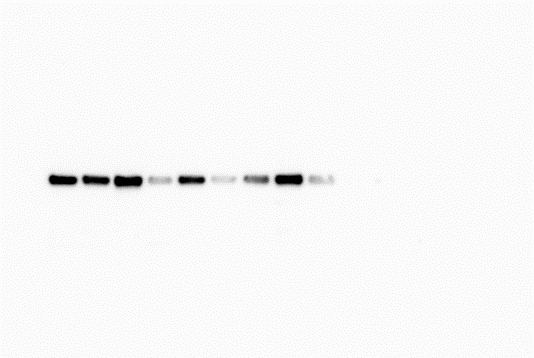

Supplement: Figure 2—source data 2. — Each lane represents a different sample, from sham mice (+) or denervated mice (−). [file elife-81996-fig2-data2.zip › Figure 2 - Source Data 1/Figure 2 - Source data 1 - TopLeft-TyrHydoxylase.png]

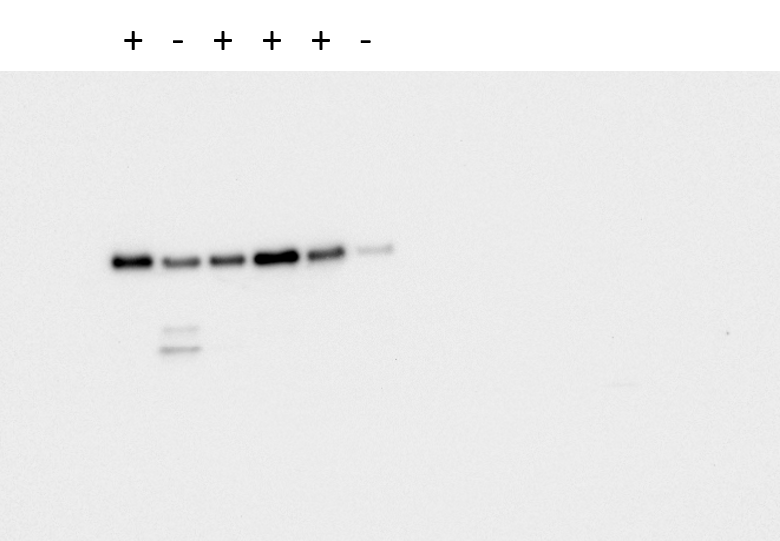

Supplement: Figure 2—source data 2. — Each lane represents a different sample, from sham mice (+) or denervated mice (−). [file elife-81996-fig2-data2.zip › Figure 2 - Source Data 1/Figure 2 - Source data 1- BottomRight-TyrHydoxylase_uncropped.png]

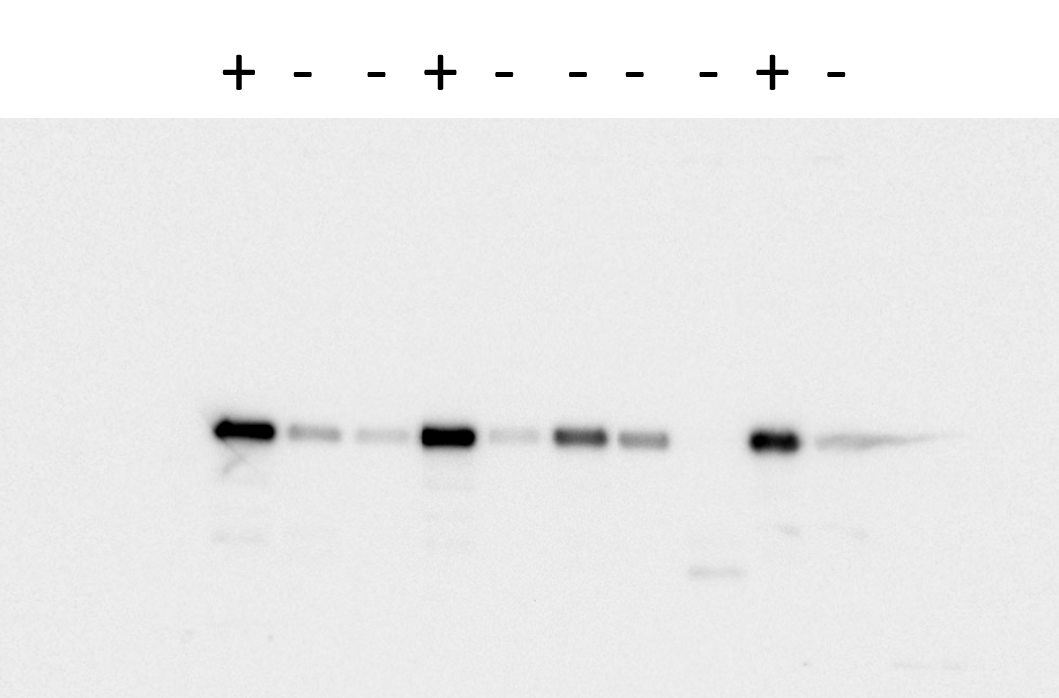

Supplement: Figure 2—source data 2. — Each lane represents a different sample, from sham mice (+) or denervated mice (−). [file elife-81996-fig2-data2.zip › Figure 2 - Source Data 1/Figure 2 - Source data 1 - TopRight-TyrHydoxylase_uncropped.png]
